# Changes in hydration structure are necessary for collective motions of a multi-domain protein

**DOI:** 10.1038/srep26302

**Published:** 2016-05-19

**Authors:** Tomotaka Oroguchi, Masayoshi Nakasako

**Affiliations:** 1 Department of Physics, Faculty of Science and Technology, Keio University, 3-14-1 Hiyoshi, Kohoku-ku, Yokohama, 223-8522 Japan; 2 RIKEN SPring-8 Center, 1-1-1 Kohto, Sayo, Sayo-gun, Hyogo 679-5148 Japan

## Abstract

Conformational motions of proteins are necessary for their functions. To date, experimental studies measuring conformational fluctuations of a whole protein structure have revealed that water molecules hydrating proteins are necessary to induce protein functional motions. However, the underlying microscopic mechanism behind such regulation remains unsolved. To clarify the mechanism, multi-domain proteins are good targets because it is obvious that water molecules between domains play an important role in domain motions. Here, we show how changes in hydration structure microscopically correlate with large-amplitude motions of a multi-domain protein, through molecular dynamics simulation supported by structural analyses and biochemical experiments. We first identified collective domain motions of the protein, which open/close an active-site cleft between domains. The analyses on changes in hydration structure revealed that changes in local hydration in the depth of the cleft are necessary for the domain motion and *vice versa*. In particular, ‘wetting’/‘drying’ at a hydrophobic pocket and ‘adsorption’/‘dissociation’ of a few water molecules at a hydrophilic crevice in the cleft were induced by dynamic rearrangements of hydrogen-bond networks, and worked as a switch for the domain motions. Our results microscopically demonstrated the importance of hydrogen-bond networks of water molecules in understanding energy landscapes of protein motions.

Water is the ‘matrix of life’ that is indispensable for every biomolecular process, such as protein folding, biomolecular interaction and protein function^1^,^2^,^3^,^4^,^5^. To reveal the role of water molecules in these processes, the understanding of the structure and modes of interaction of water at the interface with biomolecules, *i*.*e*. hydration structure, is very important^3^,^4^. Experimental studies measuring mean-square atomic displacements of a whole protein structure have revealed that at least a single layer of hydration water molecules is necessary to induce large-amplitude collective protein conformational motions, which are functionally essential^6^,^7^,^8^,^9^,^10^,^11^,^12^,^13^. Molecular dynamics (MD) simulation studies have also indicated that changes in hydration structure add certain energy barriers to the energy landscapes of proteins, and thus result in anharmonic collective motions^14^,^15^,^16^.

Collectively, the current literatures postulate that protein functional motions couple with changes in the hydration structure. However, the underlying atomic view for such coupling is still unclear, as the experimental studies so far have provided little microscopic information regarding changes in the hydration structure. The simulation studies have focused mainly on protein motions themselves and have conducted little analyses on coupling between protein motions and changes in hydration structure, owing to the difficulty of tracking random motions of individual water molecules on complex protein surfaces.

To investigate the engagement of water molecules in protein functional motions, multi-domain proteins are good targets because it is obvious that water molecules between domains are important for collective domain motions (Fig. 1a). In addition, most proteins are composed of multiple domains, and it is well known that domain motions are general functional motions^17^,^18^,^19^. In this study, we have used hexameric glutamate dehydrogenase (GDH) as a target protein (Fig. 1b). Each subunit of GDH comprises a nucleotide-binding domain (N-domain) and a core domain (C-domain) for hexamer formation (Fig. 2a). A large cleft between the two domains serves as the active site to which substrate binds^20^,^21^. Cryogenic X-ray crystal structure analyses of unliganded GDH from a hyperthermophilic archaeon have revealed several metastable conformations in N-domain motion to open/close the active-site cleft^22^,^23^. More importantly, the hydration structures visualized in each trapped conformation suggest a cooperative variation of hydration in coupling with the domain motion.

To understand the role of water molecules in functional motions of a multi-domain protein, here we study the spontaneous domain motions of unliganded GDH in solution through a 200-ns molecular dynamics (MD) simulation in explicit aqueous solvent. The domain motion observed in the MD simulation agreed well with the conformational variety observed in the crystal structures. The amplitude of the domain motion in the simulation also agreed with the conformational variety of domains observed in the surface topography of GDH crystals measured by atomic force microscopy (AFM). To enable the visualization of changes in hydration structure along with those domain motions, we reduced irrelevant random motions of water molecules in bulk using solvent density maps^24^ calculated within a short time window. We found strong correlation between the variations of local hydration structures in the depth of the active-site cleft and the domain motion. In particular, ‘wetting’/‘drying’^25^,^26^,^27^ at a hydrophobic pocket and ‘adsorption’/‘dissociation’^4^ of a few water molecules at a hydrophilic crevice were induced by dynamic rearrangements of hydrogen-bond networks among water molecules and protein atoms, and work as a switch for the open/closure motion of the domains.

## Results

### Molecular motions observed in MD simulation

**Figure 2:**
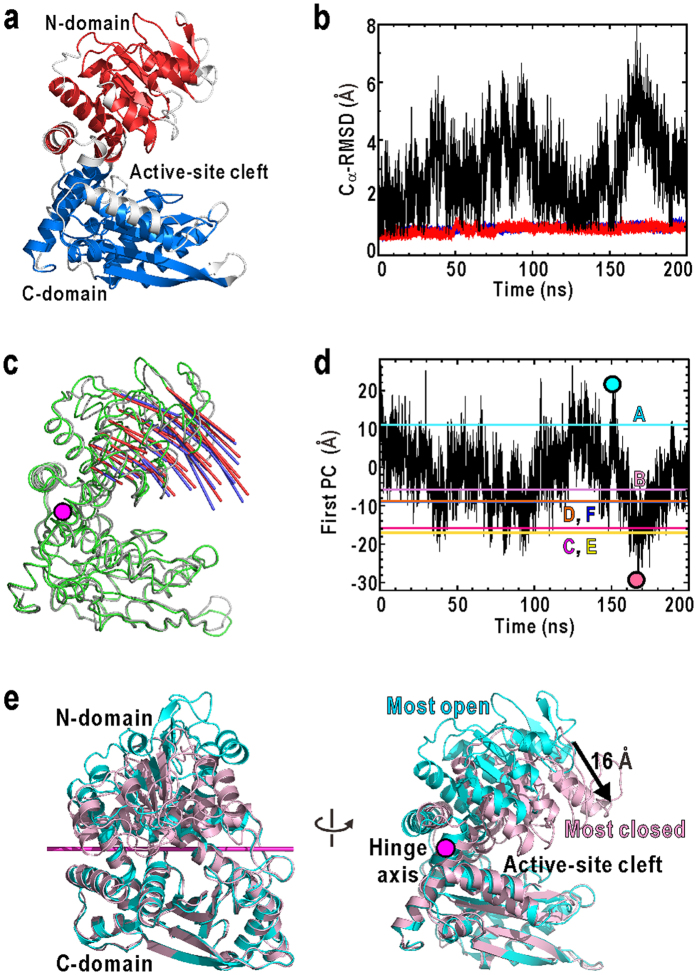
Molecular motions of GDH observed in the MD simulation. (**a**) The structure of subunit A in the crystal structure^22^,^23^. Residues are coloured by the definitions of N-domain (red, residues 24–44, 51–77, 81–112, 119–164, 171–177, 348–359, and 399–417) and C–domain (blue, residues 189–205, 211–244, 248–264, 276–295, 306–331, 335–346, and 369–388), which were used in the calculation of Cα-RMSD. In the above definitions of the domains, the fluctuating helices and the loop regions (white) were excluded, because the random fluctuations of these structural parts were trivial for the assessments of the stability of each domain structure. (**b**) Time courses of the Cα-RMSD of N- (red lines) and C-domains (blue lines) of subunit A. Black lines show the time course of the Cα-RMSD of N-domains when superimposing the C-domains. The data for all subunits are shown in Supplementary Fig. S1b. (**c**) The N-domain motion along the first PC axis in the MD trajectory (red arrows with amplitude drawn at 8σ level) is compared with that in the 6 subunits of the crystal structure (blue, 10σ) on the average structures (green (MD) and grey (crystal)). A magenta stick indicates the hinge axis of the first PC, determined by program DyDom^46^. (**d**) Time course of the domain movements of subunit A along the first PC in the MD trajectory. The projections of subunits A-F in the crystal structure onto the PC axis are indicated by coloured lines. The time courses of the movements of all subunits are shown in Supplementary Fig. S1c. The most open (cyan symbol) and the closest (pink) conformations in (**d**) are illustrated in (**e**).

During the 200-ns simulation, the whole system showed little drifts of the total energy. The structures of both N- and C-domains remained rigid, *i.e*. the root-mean square displacements of Cα-atoms from the initial structure remained around 1 Å for all subunits (Fig. 2b for subunit A and Supplementary Fig. S1b for all subunits). In contrast, when superimposing only the C-domains, the Cα-RMSD of the N-domain varied up to 8 Å. These results indicated the rigid-body motions of N-domain against C-domain in each subunit.

The rigid-body motions of the N-domain relative to the C-domain were analysed using principal component analysis^28^ (PCA). The most major motion identified by PCA, *i.e*., the first principal component (PC) (Fig. 2c), accounted for 72% of the total fluctuations of the N-domain motion. This motion is a hinge motion that drives N-domain by approximately 16 Å at the tip (Fig. 2e), and stochastically opens/closes the active-site cleft as seen in the time course of the movements along the first PC (Fig. 2d). There is little correlation in the time course of the first PCs among all subunits (Supplementary Fig. S1c), indicating that the N-domain of each subunit moves independently from the other.

To compare the simulated N-domain motion with the structural variations observed in the crystal structure (Supplementary Fig. S1d), the subunit conformations in the crystal were also analysed by PCA. The first PC axis in the simulation (red arrows in Fig. 2c) agreed well with that of the crystal structure (blue arrows in Fig. 2c) with a correlation coefficient of 0.98. Conformations equivalent to the subunits of the crystal structure were frequently observed in the MD trajectory (coloured lines in Fig. 2d). Therefore the N-domain motions observed in the MD were consistent with the conformational variety of N-domain in the crystal structure. These results indicate that the subunits of GDH intrinsically undergo molecular motions to open/close the active-site cleft even without substrates. In fact, regarding the domain arrangement, the most closed conformation observed in the present simulation (the projection on the first PC = −30 Å) was almost close to the substrates-bound conformation observed in the crystal structure of GDH from Bovine^29^ (the projection on the first PC = −38 Å).

### Conformational variety observed through AFM

**Figure 3:**
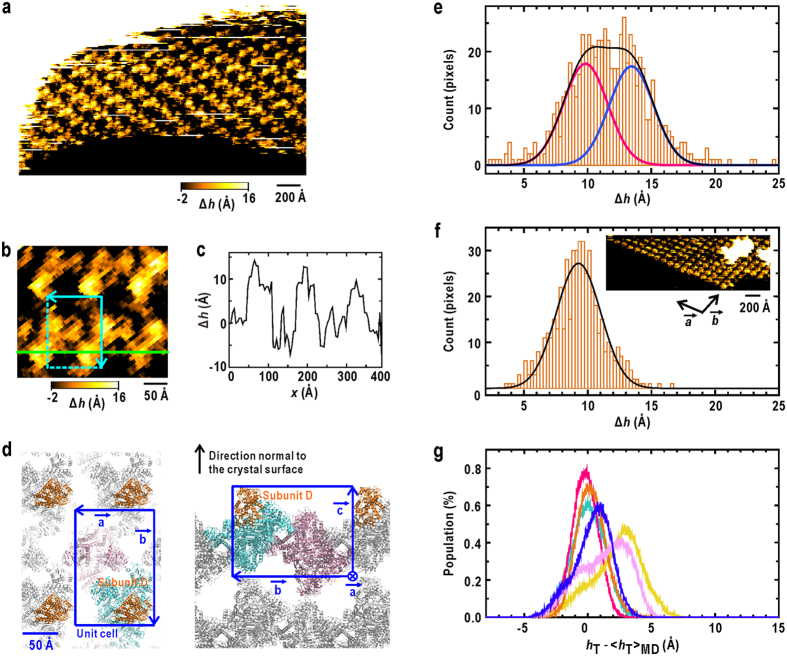
AFM measurements on the surface of GDH crystals. (**a**) A topography after the tilt-correction of the crystal surface against the horizontal movement of the piezo-scanner. ∆*h* is the height from the average plane of the image. (**b**) A magnified view of the topography shown in (**a**). The cyan box is a unit cell. (**c**) The height profile along the green line in (**b**) is shown. (**d**) The illustrations showing molecular packing mode of GDH in the monoclinic crystal^22^,^23^, viewed normal to the *a*-*b* plane (left panel) and along the *a*-axis (right panel). The blue boxes are unit cells. (**e**) The height distribution (orange bars) of N-domain of subunit D from the AFM surface topography for GDH crystals. The distribution is approximated by the sum (black curve) of two Gaussians (red and blue curves) with the standard deviation values determined from the AFM topography for the surface of haemoglobin crystals. (**f**) The height distribution (orange bars) of subunits of haemoglobin in the AFM topography (inset). The black curve is a Gaussian with the standard deviation of 3.4 Å approximating the height distribution. (**g**) Simulated height distribution of N-domain from the MD trajectory of each subunit. The colouring scheme is the same as in Fig. 1b. *h*_T_ is the height of the centroid of residues 277–287 at the tip of N-domain of subunit D along the direction normal to the crystal surface (Supplementary Note 1 and Supplementary Fig. S3). 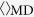 is the average over the MD trajectories. The total sum of distributions is approximated by two Gaussians.

The conformational variety of GDH subunits was also assessed through the AFM measurements regarding the molecular heights of N-domains on the surfaces of GDH crystals (Fig. 3). Through comparing the crystal structure and the unit cells on the crystal surfaces (Fig. 3d), the N-domains of subunits D were found to be free from crystal contacts and protruded from the surface with the largest heights. Therefore, the N-domains of subunits D are suitable as an experimental reference to examine the magnitude of domain motions in the MD simulation. Indeed, the height profile on the AFM topography (Fig. 3c) shows the variety in the molecular heights of these protruding subunits, which would reflect the variety in the position of N-domain relative to that of C-domain.

From 133 images of subunit D selected from surface topographies of GDH crystals (Fig. 3a and Supplementary Fig. S2b), we obtained the height distribution of N-domain appeared as a trapezoid shape in the range of approximately 5–20 Å (Fig. 3e). As a reference estimating the influences of the Brownian fluctuations of cantilever probe on the surface profile, we also measured surface topography of haemoglobin crystal^30^, the subunits of which are more rigid than those of GDH. The height distribution of haemoglobin subunits varied in the width of 10 Å, and was approximated by a single Gaussian with a standard deviation of approximately 3.4 Å (Fig. 3f). The width of the height distribution of N-domains of GDH was clearly wider than that of haemoglobin subunits, and therefore indicates the conformational variations of GDH subunits. The height distribution along the direction normal to the crystal surface revealed at least two conformational states of N-domain (Fig. 3e). The maxima of their height distributions were separated by 3.6 ± 0.3 Å with the fluctuations of approximately 3.4 Å.

To compare the domain motion in the MD trajectory with the AFM results, we superimposed the C-domains of subunits in all trajectories onto the C-domain of the most exposed subunit in the unit cell of crystal surface (Supplementary Note 1 and Supplementary Fig. S3). Then, the simulated height distributions were calculated for six subunits (Fig. 3g). The N-domain motions in the MD simulation superimposed onto the crystal surface were classified roughly into two conformational states, the maxima of which were separated by 2.9 ± 0.1 Å. Therefore, there was the qualitative similarity in the amplitude of the height distribution of the N-domains of the most exposed subunit on crystal surface between the MD simulation and AFM.

These agreements among the simulation, the crystal structure and the AFM measurements indicate that, in solution, GDH spontaneously undergoes the open/close N-domain motion, which is necessary to clamp substrates. However, the differences were also observed in the population of these open and closed conformations between the MD simulation and the AFM measurements. These differences would be due to the insufficient sampling of the subunit conformations of GDH in the MD simulation, and also due to the difference in solution condition between the MD and the AFM experiments.

### Two regions displaying both conformational and hydration changes along with N-domain motion

**Figure 4:**
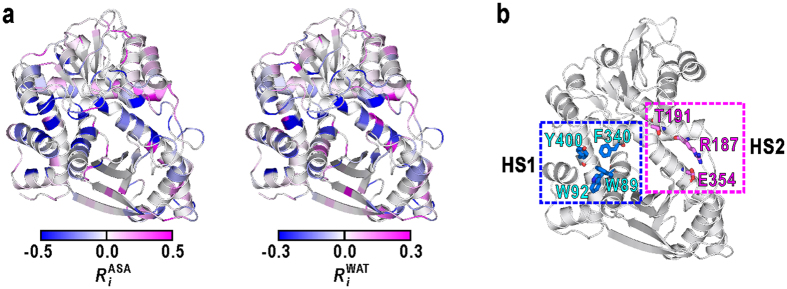
Regions displaying conformational and hydration changes along with N-domain motion. (**a**) Regions displaying conformational and hydration changes along with N-domain motions. Each residue is coloured according to changes in ASA (*R*_*i*_^ASA^) and hydration (*R*_*i*_^WAT^) in the closed conformation when compared with the open conformation. A negative value of *R*_*i*_^ASA^ indicates a decrease in the ASA of the *i*-th residue in the closed conformation when compared with the open conformation. A negative value of *R*_*i*_^WAT^ indicates a decrease in the number of water molecules around the *i*-th residue. (**b**) The locations of two clusters of amino acid residues, HS1 and HS2.

To identify regions undergoing conformational and hydration changes along with the domain motion, the changes in the solvent-accessible surface area (ASA) and the number of hydration water molecules of each residue from the open to closed conformations were monitored by *R*_*i*_^ASA^ and *R*_*i*_^WAT^, respectively (Fig. 4a). Two clusters of amino-acid residues, HS1 and HS2, displayed significant decreases in both ASA and hydration (Fig. 4b).

One cluster, hereafter designated as hydration site 1 (HS1), comprises a hydrophobic pocket with an approximate size of 7 Å. The pocket is formed by the lower jaw of residues W89 and W92, and the upper jaw of F341 and Y400. The other cluster, designated as hydration site 2 (HS2), has a columnar hydration crevice with a length of 10 Å and a width of 6 Å, formed by hydrophilic residues R187, T191, and E354. Structurally, both HS1 and HS2 are each approximately 7 Å apart from the hinge axis for the N-domain motion, which lies near and parallel to a long α-helix forming the backbone of the active-site cleft (Fig. 2e). Therefore, the conformational changes of these two sites can be amplified to the N-domain motion as shown by the following analyses.

### Conformational and hydration changes at the hydrophobic pocket HS1

**Figure 5:**
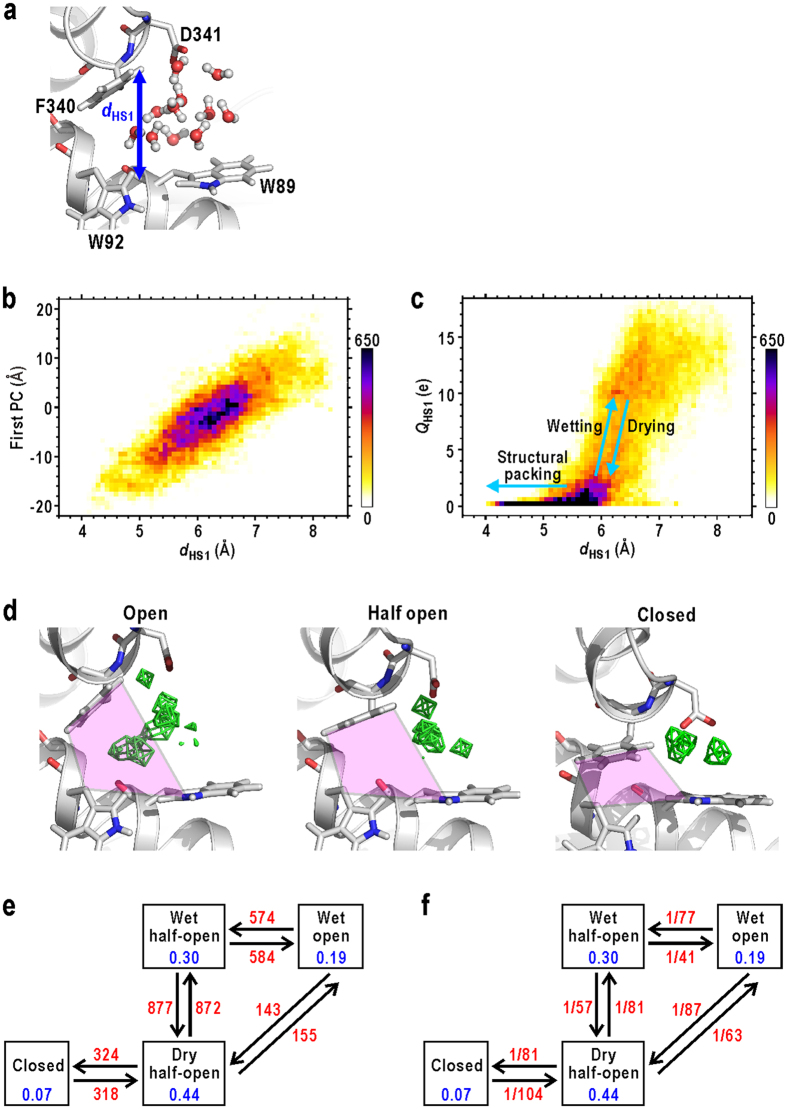
Conformational and hydration changes at HS1. (**a**) An illustration defining *d*_HS1_. (**b**) A heat map displaying the dependence of the N-domain motion, represented by the first PC, on *d*_HS1_. The shown map is for the data of subunit A, and that for the merged data of all subunits is shown in Supplementary Fig. S9a. (**c**) A heat map displaying the relation between *d*_HS1_ and *Q*_HS1_ of subunit A. The map for the merged data of all subunits is shown in Supplementary Fig. S9b. (**d**) Solvent density maps (green fishnets) in the open, half-open, and closed conformations of HS1 contoured at 1.5 times the averaged bulk solvent density. Regions used to monitor *Q*_HS1_ are coloured in pink. The frequencies (**e**) and rate constants (**f**) between the conformational and hydration states at HS1 (red coloured characters). The unit for rates is ps^−1^. Blue coloured characters are the population of each state.

Major conformational changes at HS1 were the open/close motion of the hydrophobic pocket. This motion could be monitored by the distance between the upper and lower jaws of the pocket (*d*_HS1_). The *d*_HS1_ is the distance between the H_δ1_ or H_ε1_ atom of F340 in the upper jaw of the active-site cleft and the midpoint of the C_δ1_ atom of W89 and the C_γ_ atom of W92 in the lower jaw. (Fig. 5a). The *d*_HS1_ stochastically varied from approximately 4.0 to 8.5 Å (Supplementary Fig. S7c), but was in concert with the first PC motion as shown by the heat map displaying the relation between these two parameters (Fig. 5b). This concert (correlation coefficient, 0.8) indicates that the hinge motion of rigid N-domain coupled with the open/close motions of the hydrophobic pocket. The structural change of the pocket by 4.5 Å in *d*_HS1_ was amplified to approximately 16 Å at the tip of N-domain apart from the hinge (Fig. 2e).

The above open-close motion of the hydrophobic pocket coupled with the hydration state of HS1. Residence of water molecules inside the pocket was monitored by the solvent density map around W89 (Fig. 5d). When HS1 was in an open conformation (*d*_HS1_ > 7 Å), solvent density peaks of a few water molecules appeared inside the pocket, *i.e*. the pocket was ‘wet’. In contrast, when HS1 was in a half-open conformation (5 < *d*_HS1_ < 7 Å), the pocket frequently changed to the ‘dry’ state, i.e., no water molecules penetrated into the pocket, despite having size large enough to allow the residence of approximately two water molecules. In the closed conformation (*d*_HS1_ < 5 Å), HS1 was tightly packed.

The hydration changes at HS1 were quantitatively characterised through the sum of the solvent densities inside the hydrophobic pocket (the pink region in Fig. 5d), *Q*_HS1_. Parameter *Q*_HS1_ monitored the sum of the solvent densities of only 1-Å cube voxels inside the hydrophobic pocket of HS1 (see Supplementary note 5 and Supplementary Fig. S7a,b for the details). The *Q*_HS1_ stochastically varied between 0 and 20 electrons (Supplementary Fig. S7d), but coupled with *d*_HS1_ as shown by the heat map displaying the relation between these two parameters (Fig. 5c).

According to the heat map, we defined the four conformational and hydration states of HS1 as follows: wet open, wet half-open, dry half-open, and closed states (see Methods for the definitions). Then, we calculated the frequencies of state changes (Fig. 5e) and their rate constants (Fig. 5f). The most popular conformation was the half open, and only in this conformation, did HS1 frequently change between ‘wet’ and ‘dry’ states. The rate of ‘drying’ was 1/57 ps^−1^, and faster than that of ‘wetting’ (1/81 ps^−1^). These rates were significantly slower than the translational relaxation time of a water molecule in bulk^31^ (approximately 5 ps). These findings imply that the ‘dry’ state is more thermodynamically stable than the ‘wet’ state.

It is also noteworthy that direct transitions between the wet-open and the closed conformations were not observed. Only after reaching the ‘dry’ half-open conformation, the pocket became the closed conformation (Fig. 5c,e). Because the empty space inside the ‘dry’ pocket might be disadvantageous for the translational entropy of water molecules^26^,^27^,^32^, the structural packing to diminish the space occurred and that brought the ‘dry’ half-open conformation to the closed one.

### Conformational and hydration changes at hydrophilic crevice HS2

**Figure 6:**
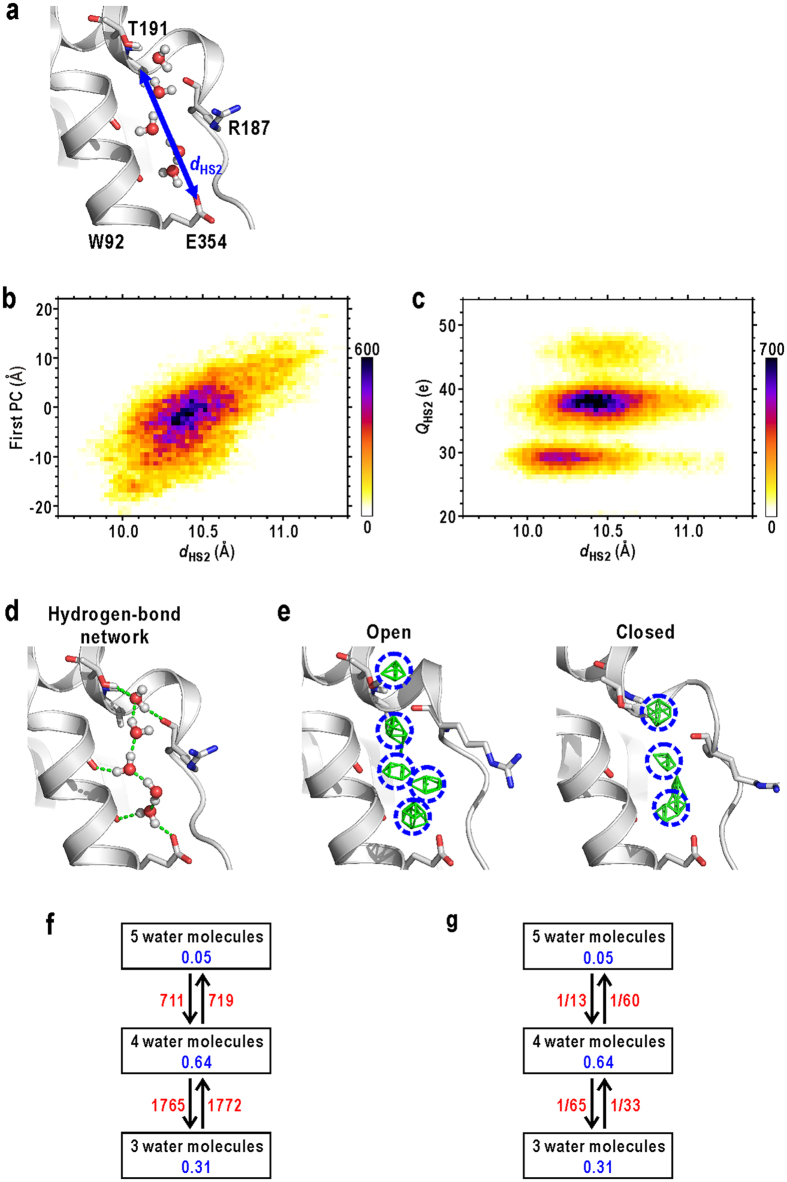
Conformational and hydration changes at HS2. (**a**) An illustration defining *d*_HS2_. (**b**) A heat map displaying the dependence of the N-domain motion, represented by the first PC, on *d*_HS2_. The shown map is for the data of subunit A, and that for the merged data of all subunits is shown in Supplementary Fig. S9c. (**c**) A heat map displaying the relation between *d*_HS2_ and *Q*_HS2_ of subunit A. The map for the merged data of all subunits is shown in Supplementary Fig. S9d. (**d**) A typical example of the H-bond network at HS2. (**e**) Solvent density maps in the open and closed conformations. Regions used to monitor *Q*_HS2_ are indicated by blue circles. (**f**) The time-course of *Q*_HS2_ in the 200-ns MD trajectory. The frequencies (**f**) and rate constants (**g**) between the conformational and hydration states at HS1 (red coloured characters). The unit for rates is ps^−1^. Blue coloured characters are the population of each state.

Next, we analysed conformational and hydration changes at HS2. The major conformational change at HS2 was the stretch/contraction of the hydrophilic crevice as monitored by the distance between the A190 C_α_ atom and the E354 O_ε1_ atom (*d*_HS2_) (Fig. 6a). The 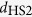 ranged from 9.5 to 11.5 Å (Supplementary Fig. S8b) and was dependent on the N-domain motion represented by the first PC (the heat map in Fig. 6b) to a lesser extent than in the case of *d*_HS1_ (correlation coefficient, 0.69). As in HS1, the small conformational changes in HS2 could be amplified to the N-domain motion, due to close location to the hinge.

The hydration structure at HS2 had columnar arrangements of three to five hydration water molecules and was stabilized by H-bond network between those water molecules and polar protein atoms (Fig. 6d,e). Therefore, the hydration structure at HS2 was dominated by the arrangements of the hydrophilic residues along HS2. The sum of the solvent electron densities in HS2, *Q*_HS2_ (Fig. 6e and Supplementary note 5), varied frequently (Supplementary Fig. S8c), but *Q*_HS2_ was almost discretized to three values (30, 40, and 50 e) as seen in the heat map displaying the relation between *d*_HS2_ and *Q*_HS2_ (Fig. 6c). Therefore the variations of the hydration structure at HS2 were characterized by ‘adsorption’/‘dissociation’ of a few water molecules with the hydrophilic crevice.

In the most popular state, HS2 with a *d*_HS2_ of 10–11 Å contained four water molecules. The hydration changes from the most popular state to minor states containing three and five water molecules occurred at a rate of 1/60 ps^−1^ and 1/65 ps^−1^, respectively. These rates were 2–4 fold slower than the rates of changes from three and five water molecules to four (1/13 and 1/33 ps^−1^, respectively) (Fig. 6g). As well as HS1, the time for changes in hydration structure at HS2 was slower than the relaxation time of water molecules in bulk. It is also noteworthy that the ‘adsorption’/‘dissociation’ of water molecules at HS2 occurred more frequently than the ‘drying’/‘wetting’ at HS1 (Figs 5e and 6f). This would be due to easier accessibility of HS2 for solvents than that of HS1.

The minimum size possible for the crevice depended on *Q*_HS2_ (Fig. 6c). For instance, when three water molecules occupied HS2, the minimum size was as small as 9.8 Å. When one additional water molecule adsorbed into HS2, the lower limit of the size increased by 0.2 Å. Therefore, the number of water molecules in HS2 restricts its minimum size and therefore correlated with the open/close motion of N-domain.

## Disucssion

In this simulation and experimental study, we found the coupling between the change in local hydration at the two hydration sites, HS1 and HS2, and the domain motion in GDH. Here we discuss the molecular mechanism of the hydration changes coupled with the domain motion based on the present results.

**Figure 7:**
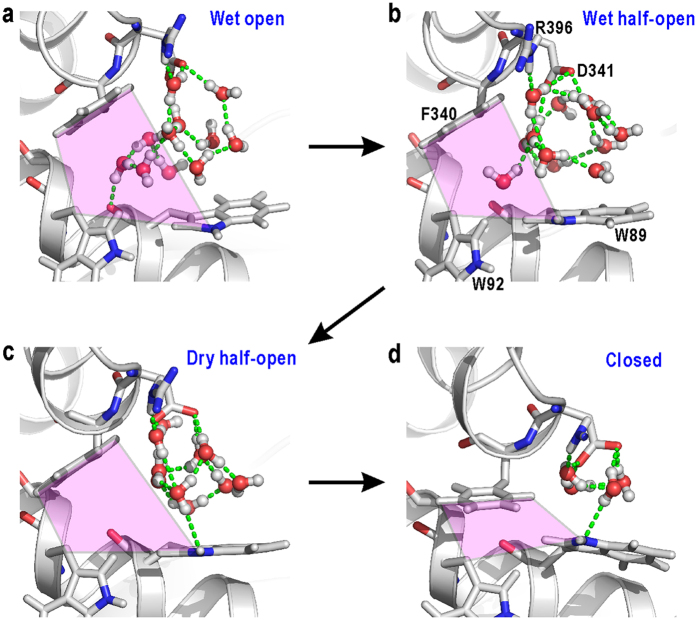
Drying and wetting processes observed at HS1. Typical examples of snapshots obtained for the wet open (**a**), wet half-open (**b**), dry half-open (**c**), and closed (**d**) conformations. Green dashed lines are H-bonds formed among water molecules in the network. Regions used to monitor *Q*_HS1_ are coloured in pink.

The analyses on the local hydration and conformational changes revealed that the open/close motions of the hydrophobic pocket at HS1 required ‘drying’/‘wetting’ in the pocket and were amplified to the N-domain motion. The mechanism of ‘drying’ in the half-open conformation of HS1 can be explained by the connectivity of the H-bond networks of water molecules formed inside and outside the hydrophobic pocket (Fig. 7). In the open conformation, HS1 was large enough for water molecules inside the pocket to contact with the H-bond network outside the pocket through two or three H-bonds (Fig. 7a). In contrast, water molecules residing in the half-open pocket interacted weakly with atoms of HS1, through van der Waals interactions only, and rarely contacted the exterior H-bond network (Fig. 7b). Once water molecules inside the pocket interacted with the exterior network, they tended to escape from the pocket to participate in the network to utilize their four H-bond arms, *i.e*., the ‘dry’ state of the pocket became thermodynamically stable (Fig. 7c). These observations indicate that ‘drying’ originated from the poor connectivity of the H-bond network inside the hydrophobic pocket. Because the expulsion of water molecules from the pocket should be an energy barrier for the domain-closure motion, the HS1 hydrophobicity inducing the spontaneous drying through the H-bond network could be important for the function of GDH.

In this regard, we measured the catalytic activity of the mutant W89F with a reduced hydrophobicity of HS1 (Supplementary Note 6 and Supplementary Fig. S11). W89F displayed the catalytic rate drastically slower than the wild type, while the substrate affinity was almost the same as the wild type (Table S1). Because the domain-closure motion clamps a substrate inside the active-site cleft for catalysis^29^,^33^, the slower catalytic rate of W89F would indicate the importance of the ‘drying’ process in HS1 for the closure motion. On the other hand, W89F mutation did not influence the affinity, because residue W89 has no direct substrate interactions.

Other studies have shown that hydrophobic clusters are often seen in functionally important regions of proteins^34^,^35^,^36^. The present study suggests how such hydrophobic clusters work in protein functional motions by using H-bond networks around them. The hydrophobicity of HS1 is well conserved among GDH from various organisms (Supplementary Fig. S12a), and this fact supports the robustness of the suggested mechanism. To monitor the ‘drying’ process at HS1 experimentally, it is necessary to measure hydration dynamics such as through the picosecond-resolved fluorescence transients^37^ of W89, which probe relaxation process of H-bond network around tryptophan residue. Furthermore, in future work, the more detailed analyses on the H-bond network and the hydrophobic surface of HS1 are necessary to elucidate the physical origin of the coupling between the ‘wetting’/‘drying’ processes and the open/close motion of the hydrophobic pocket.

**Figure 8:**
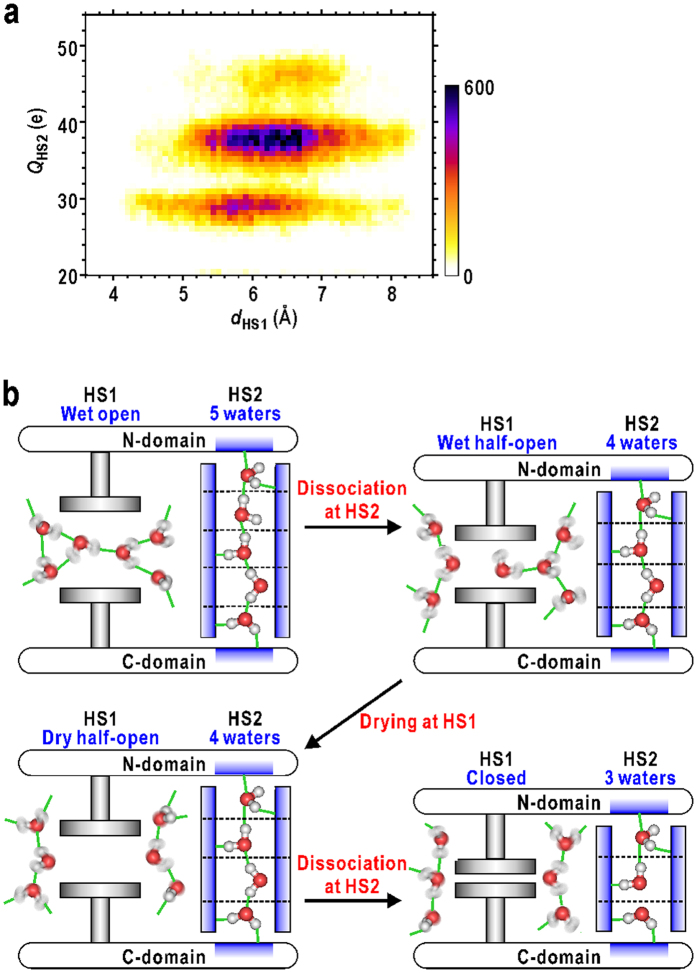
The mechanism of the hydration-regulated domain motion in GDH. (**a**) A heat map displaying the relation between *d*_HS1_ and *Q*_HS2_. The shown map is for the data of subunit A, and that for the merged data of all subunits is shown in Supplementary Fig. S9e. (**b**) A schematic illustration explaining the mechanism of the hydration-regulated domain motion in GDH. The hydrophobic and hydrophilic surfaces are coloured in grey and blue, respectively.

The present analyses on the MD trajectory also revealed that the number of water molecules in HS2 restricts the minimum size of the hydrophilic crevice and therefore correlated with the open/close motion of N-domain (Fig. 6c,e). The observed conformational and hydration changes at HS2 also coupled with those at HS1. Because HS1 and HS2 are located near the hinge axis and the two domains are rigid, the minimum size of HS1 is likely to be affected by the size of HS2 depending on the number of water molecules (Fig. 8a). For instance, when HS1 was in a closed conformation (*d*_HS1_ < 5 Å), HS2 was most probably filled with three water molecules rather than four or five. The half-open conformation of HS1 (5 < *d*_HS1_ < 7 Å) appeared frequently when HS2 was filled with four water molecules. When five water molecules filled HS2, structural packing of HS1 could not occur (*d*_HS1_ > 5 Å). Therefore, the ‘dissociation’ of water molecules from HS2 is necessary for the complete structural packing of HS1. A longer column of water molecules at HS2 more effectively hinders both the structural packing at HS1 and the domain-closure motion.

The stable hydration of a hydrophilic protein surface requires the arrangement of H-bond donors and acceptors in a tetrahedral H-bond geometry of water molecules^38^. Therefore, the hydrophilic crevice of HS2 only allows the stepwise ‘adsorption’/‘dissociation’ of water molecules, which is accompanied by changes in the total number of H-bonds. This scheme of hydration in HS2 is quite different from HS1, where water molecules are in van der Waals contacts with aromatic side chains. In this way, the hydration at HS2 works as a ‘plasticizer’ for protein motions. As well as HS1, the amino-acid residues forming HS2 are well conserved among GDH from various organisms (Supplementary Fig. 12b).

Considering these results of the structural and hydration dynamics in HS1 and HS2 along with the N-domain motion, the local conformational changes in these two sites accompanying ‘wetting’/‘drying’ and ‘adsorption’/‘dissociation’ of water molecules, respectively, are one of essential factors inducing the stochastic N-domain motion necessary to clamp the substrates to be catalysed (Fig. 8b). Hydrophobic HS1 acts as an actuator smoothly driven by the penetration of hydration water molecules and by the structural packing of the ‘dry’ pocket. In contrast, hydrophilic HS2 acts as a cylinder that changes its length through the stepwise adsorption/dissociation of hydration water molecules. In addition, the conformation/hydration states of HS1 control those of HS2, and *vice versa*. The rigidity of the N-domain and the α-helix near the hinge axis is probably essential for e bidirectional communication between HS1 and HS2. The small conformational energy barrier for the structural changes from the open to closed conformations (Supplementary Note 7 and Supplementary Fig. S13) allow the hydration changes at HS1 and HS2 to have a profound impact on the large domain motion. Therefore, the motions of the two hydration sites collectively drive the stochastic motion of the N-domain. The similar concerted changes in hydration structures along with protein structural changes have been also observed for other proteins by cryogenic X-ray crystal structure analyses^4^,^39^.

In both hydration sites, the tetrahedral arms of H-bonds of water molecules are essential for hydration structure changes. In hydrophobic HS1, the H-bond arms are used for water molecules to escape from the pocket through contact with the exterior H-bond network. In hydrophilic HS2, the arms act as glue or lubricant for the association/dissociation of water molecules. Therefore the H-bond network of water molecules plays an essential role in the regulation of protein motions. The revealed role would exemplify how the tetrahedral H-bond geometry of water molecule works in biological processes.

In conclusion, our present study using combination of molecular dynamics simulation with experiments microscopically demonstrated that water molecules hydrating protein surfaces are deeply concerned with functional motions of a multi-domain protein, GDH. Cooperative changes in hydration structure in the cleft between domains were induced by dynamic rearrangements of a hydrogen-bond network among water molecules, and worked as a switch for domain-closure motion, which is necessary to clamp substrates. Our results show the importance of water molecules in understanding energy landscapes of protein functional motions, and therefore should be important for design and engineering of protein functions.

## Methods

### MD simulation

We prepared an atomic model of a GDH molecule (the accession code of the Protein Data Bank: 1EUZ^23^) immersed in a water box with the dimensions of 144 × 144 × 160 Å^3^ (Supplementary Fig. S1a). Under periodic boundary conditions, the dimensions were wide enough to separate GDH from its periodic images by more than 40 Å. The system contained 39,136 protein atoms, 94,799 TIP3P water molecules^40^ and 12 Na^+^ ions to neutralize the system. The MD simulation was conducted at 293.15 K using the MARBLE^41^ software and the CHARMM27 force-field^42^. The electrostatic interactions were treated by using the particle-mesh Ewald method^43^. A symplectic integrator for rigid bodies^44^ was used with a time step of 2 fs.

The system was first subjected to an energy minimization of 20,000 steps. After that, to equilibrate the solvent region, we conducted a 1.2-ns NPT run^41^,^44^ at 293.15 K and 1.0 atm, under the harmonic restraint for protein atoms with the force constant of 1.0 kcal∙mol^−1^∙Å^−2^. The size of the system became 141 × 141 × 157 Å^3^. Finally, a production run of 200 ns was conducted under NPT condition without restraints. The coordinates of all atoms were saved at every 1 ps. The simulation was conducted by using 2,048 cores of SPARC64^TM^ IXfx (1.848 GHz per core) in FX10 Supercomputer System of the Information Technology Center at the University of Tokyo.

### Principal component analyses

We applied the PCA^28^ to characterize the global conformational fluctuations of the GDH subunits observed in the 200-ns MD. We defined two groups composed of several secondary structure elements in each domain. One group comprises Cα-atoms of residues 27–44, 51–75, 82–180, 340–359, and 398–419 composing the C-domain, which were used for superimposing subunits. The other is a group of Cα-atoms of residues 214–218, 241–244, 247–250, 291–294, 313–315, and 336–338 of the N-domain, which was used for the PCA calculation. As a rigid part in the N-domain, we used only the core region composed of the Rossmann fold^45^ in this calculation.

The first PC motion was also analysed by program DyDom^46^. In detail, the most open and closed conformations were first identified according to the projection values of the snapshots on the first PC. Then, the DynDom analysis for these conformations allowed us to identify the hinge axis of the first PC motion, and to define dynamic domains and hinge-bending region^47^ (Supplementary Note 7 and Supplementary Fig. S13).

The conformational variety among the six subunits in the crystal structure^22^,^23^ (Supplementary Fig. S1d) were also quantitatively analysed by using the PCA. The definitions for the domains are the same with those used for the PCA of the MD trajectory. The conformational change along the first PC axis accounts for 98% of the observed conformational variance among the six subunits.

### Mapping of conformational and hydration changes in domain motion

We here introduced parameter *R*_*i*_^ASA^ to evaluate quantitatively and systematically the structural packing of the *i*-th residue between the open and closed conformations, defined as:


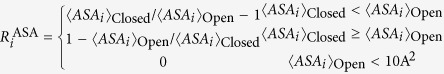


where *ASA*_*i*_ is the accessible solvent area (ASA) of the side chain of the *i*-th residue in a snapshot. ASA is calculated by using the analytical formula of ASA^48^. 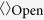 and 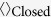 represent averages over the snapshots classified as open (projection value on the first PC axis > 15 Å) and closed (projection value on the first PC axis < −15 Å) conformations, respectively. Because the residues with a 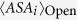 of less than 10 Å^2^ are buried inside the protein structure, they were excluded from the calculations. A negative value of *R*_*i*_^ASA^ indicates a decrease in the ASA of the *i*-th residue in the closed conformation when compared with the open conformation.

The other parameter, *R*_*i*_^WAT^, was introduced to monitor the hydration structure changes around the *i*-th residue between the open and closed conformations regarding the number of water molecules. *R*_*i*_^WAT^ is defined as:


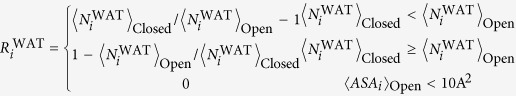


where *N*_*i*_^WAT^ is the number of water molecules within 3.5 Å from any side-chain atom of the *i*-th residue. Like in the case of *R*_*i*_^ASA^, the residues buried inside the protein structure were excluded from the calculations. A negative value of *R*_*i*_^WAT^ indicates a decrease in the number of water molecules around the *i*-th residue in the closed conformation when compared to the open conformation. The values of *R*_*i*_^ASA^ and *R*_*i*_^WAT^ from the 200-ns trajectories of all subunits are plotted in Fig. 4a.

### Calculation of solvent density map

The solvent density map was calculated from the MD trajectory by counting the number of electrons of atoms in water molecules visiting 1-Å cube voxels, which were defined to divide the simulation system^24^. High solvent density means that water molecules frequently visit and/or reside in that voxel. The time window used for the calculation was 50 ps, which was sufficiently long to reduce the noisy densities in the disordered bulk-solvent region and visualise the hydration structures (see Supplementary note 2 and Supplementary Fig. S4).

For the calculation, a set of amino-acid residues was used for the superimposition of the hydrated groups to reduce the smearing of the solvent density map caused by the translational and rotational movements of the GDH molecule. The translation vector and rotation matrix obtained from this superimposition were applied to all atoms in the simulation box, and then the map was calculated as described in the above paragraph. The α-helix including W89 (residues 87–96) was used for the superposition in the calculation of the solvent density map around HS1. For the solvent density map around HS2, the α-helix including this residue (residues 349–358) was used for the superposition. Prior to the analysis, we confirmed that solvent density maps reproduced the hydration structures found in the crystal structure (see Supplementary note 3 and Supplementary Fig. S5).

The time evolution of the solvent density map was calculated by shifting the time window of 50 ps at 1-ps intervals. For every time window, the average protein structure was also calculated. From the solvent density maps and average protein structures, we calculated the time courses of four parameters, *d*_HS1_, *Q*_HS1_, *d*_HS2_ and *Q*_HS2_. The calculation details about these parameters are described in Supplementary note 5.

### Rate constants of conformational and hydration changes

For the calculation of the rate constants of the ‘wetting’ and ‘drying’ processes occurring in the half-open conformation of HS1, we first classified every snapshot in the MD trajectory into the four conformational and hydration states by using the parameters *d*_HS1_ and *Q*_HS1_ as follows: wet open (*d*_HS1_ > 7 Å and *Q*_HS1_ > 8 e), wet half-open (5 < *d*_HS1_ < 7 Å and *Q*_HS1_ > 8 e), dry half-open (5 < *d*_HS1_ < 7 Å and *Q*_HS1_ < 4 e), and closed (*d*_HS1_ < 5 Å and *Q*_HS1_ < 4 e) states. When a snapshot was in the boundary between any pair of states, the snapshot was classified into the state temporally nearest to the snapshot.

Afterward, we calculated the frequency distribution regarding the residence time, *t*_res_, of more than 5 ps for each state. The calculated frequency distribution for the neighbouring states can be described by the survival probability function^49^, *P*_AB_(*t*_res_). *P*_AB_(*t*_res_) provides the probability that a conformation in state A at *t* = 0 remains in state A before changing to state B at *t* = *t*_res_. *P*_AB_(*t*_res_) was fitted by the function of 1 ‒ exp(−*t*_res_/τ_AB_), where 1/τ_AB_ is the rate constant for turning from state A to B, and *P*_AB_(0) equals to 1 (Supplementary Fig. S10). The frequencies and the rate constants between adjacent states are displayed for HS1 in Fig. 5e,f and for HS2 in Fig. 6f,g.

### Sample preparation

The purification of GDH samples were carried out as reported previously^21^,^22^. In this section, the procedures are briefly described. The expression vector pOTB96 carrying the wild-type GDH gene was described previously^21^. The vector for the W89F mutant was produced by a PCR-based site-directed mutagenesis using the QuikChange Site-Directed Mutagenesis Kit (Agilent Technologies) with a primer set (5′CTCGCTACCTTCATGACCTGGAA3′ and 5′TTCCAGGTCATGAAGGTAGCGAG3′). The expressed GDH proteins were roughly purified by heat treatment at 353 K for 10 min and further purified using HiTrap Q HP anion-exchange, HiTrap Phenyl HP hydrophobic-interaction, RESOURCE PHE hydrophobic-interaction, and RESOURCE Q anion-exchange chromatography columns (GD Healthcare Life Sciences) sequentially. Then, the samples were demineralized by dialysis and, finally, purified by Matrex Gel Red A affinity chromatography (Amicon Co.).

### Measurements of surface topography of GDH crystal by AFM

The crystallization of GDH samples were carried out as reported previously^22^,^50^. AFM images of GDH crystals were recorded using SPI4000/SPA400 (SII Nano Technology, Japan) equipped with a piezo scanner and a cantilever holder for liquid. Cantilevers of 200 μm (Olympus, Japan) were set to the cantilever holder. GDH crystals soaked in stabilization buffer, containing 1.7 M lithium sulphate and 0.1 M sodium acetate (pH 4.5), were placed inside a custom-made sample chamber sealed with a thin polymer film to keep the saturated vapour pressure. After the thermal equilibrium of the sample chamber, AFM topographies were collected in the contact mode with a minimum contact force of less than 100 pN and a scan frequency of 0.5–1.0 Hz. The typical scan area was 250 × 250 nm with 512 raster pixels and 256 step pixels.

The raw surface images were processed to get the height distribution of N-domains free from crystal contacts on crystal surfaces through the correction of the inclination of the crystal surface against the scanner, the assignment of unit cells in surface topography and measurement of height distribution of the N-domain motion. The details for the data processing are described in Supplementary note 1 and Supplementary Fig. S2 together with the details regarding the calculation of height distribution of N-domain from the MD trajectory (Supplementary Fig. S3).

## Additional Information

**How to cite this article**: Oroguchi, T. and Nakasako, M. Changes in hydration structure are necessary for collective motions of a multi-domain protein. *Sci. Rep*. **6**, 26302; doi: 10.1038/srep26302 (2016).

## Supplementary Material

Supplementary Information

## Figures and Tables

**Figure 1:**
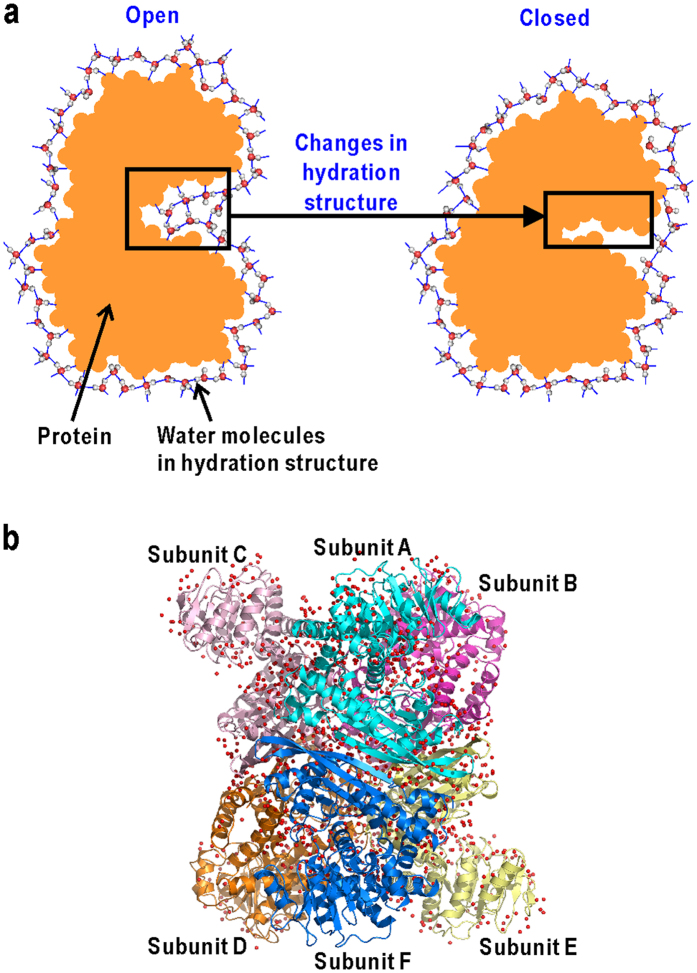
A multi-domain protein as a target system in this study. (**a**) Schematic illustration of coupling between changes in hydration structure and domain motion of multi-domain protein. (**b**) The crystal structure of GDH^22^,^23^. The subunits are coloured differently (cyan, pink, magenta, orange, yellow, and blue for subunits (**A**–**F**), respectively).
