# Supplementary material for: Changes in hydration structure are necessary for collective motions of a multi-domain protein
Source: Sci Rep. 2016 May 19;6:26302. doi: 10.1038/srep26302 (PMC4872039; doi:10.1038/srep26302)
Supplement: Supplementary Information [file srep26302-s1.pdf]

Supplementary Information for

"Changes in hydration structure are necessary for collective motions of a multi-domain protein"

Tomotaka Oroguchi [a,b] and Masayoshi Nakasako [a,b,*]

[a]Department of Physics, Faculty of Science and Technology, Keio University,

3-14-1 Hiyoshi, Kohoku-ku, Yokohama, 223-8522 Japan

[b]RIKEN SPring-8 Center, 1-1-1 Kohto, Sayo, Sayo-gun, Hyogo 679-5148 Japan

[*] To whom correspondence should be addressed. Phone: +81-45-566-1713. Fax: +81-45-566-1672.

E-mail: nakasako@phys.keio.ac.jp.

Supplementary Figures

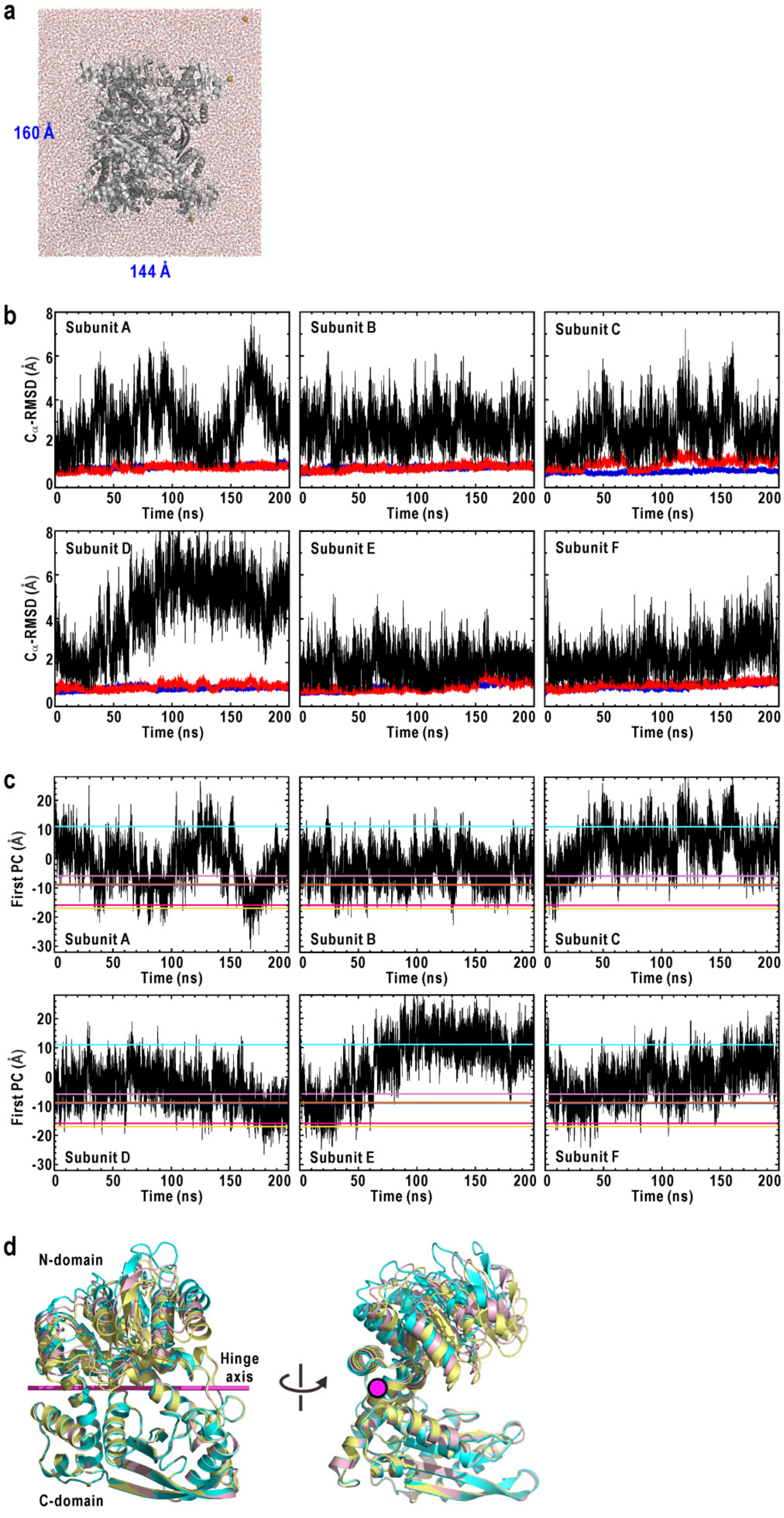

Supplementary Figure S1. Details of the N-domain motion of GDH. (**a**) The system prepared for the MD simulation of GDH with explicit water molecules. (**b**) Time courses of the Cα-RMSD of the N- (red lines) and C-domains (blue lines) in each subunit. Black lines show the time course of the Cα-RMSD of N-domains when superimposing the C-domains. (**c**) Time courses of the movements along the first PC in the 200-ns MD trajectories of the six subunits. The zero value of the first PC corresponds to the structure averaged over the trajectories of the six subunits. The cyan, pink, magenta, orange, yellow, and blue lines represent the projection values of the conformations of the six subunits A-F from the crystal structure[22,23], respectively, onto the first PC axis. (**d**) Conformational variety among subunits A, C and E in the crystal structure of GDH. The hinge axis of the motion from the open (subunit A) to the closed conformations (subunit E), which is determined by program DynDom[46], is represented by a magenta stick. The hinge axis of the N-domain motion is shown as a magenta circle.

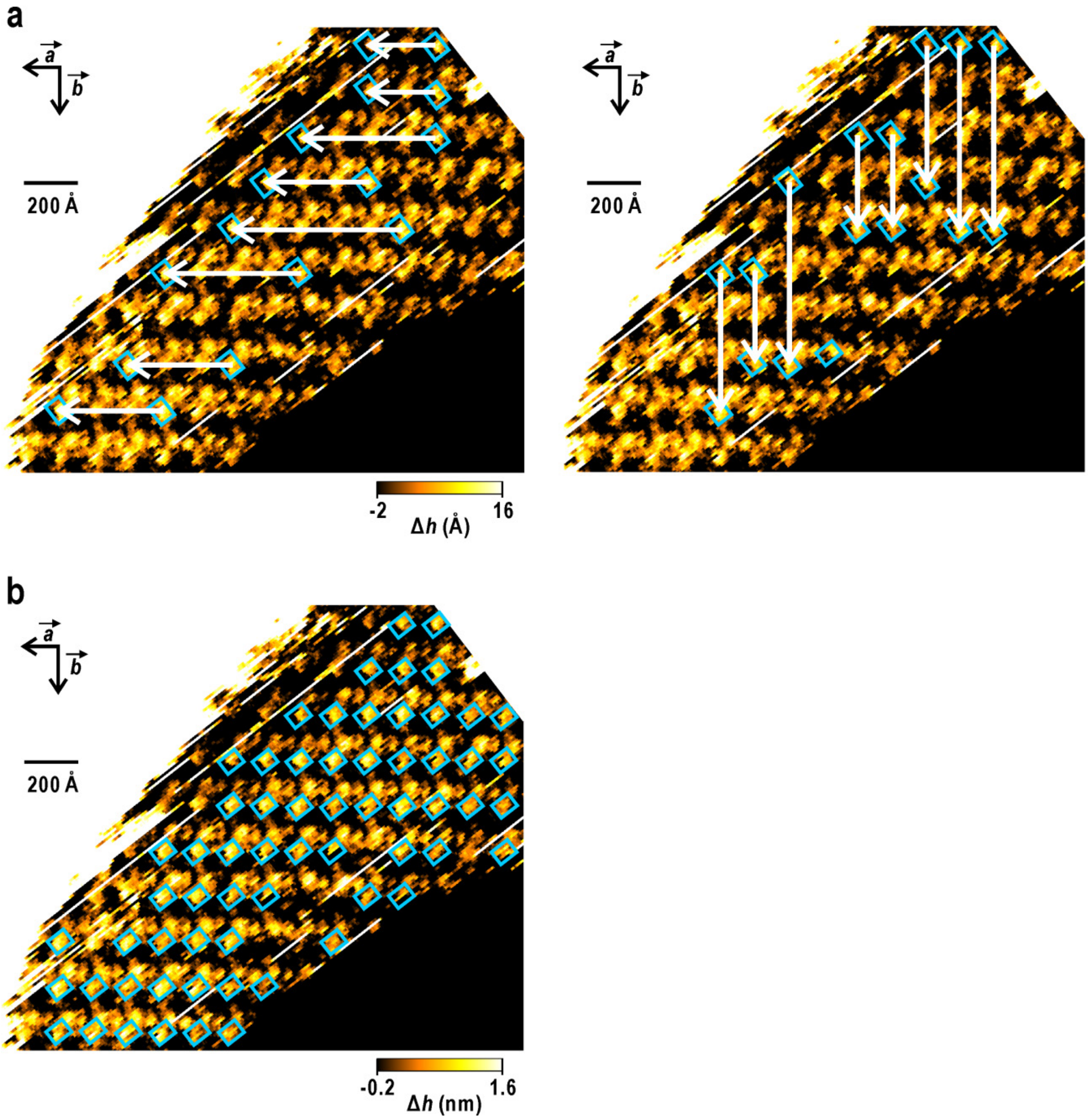

Supplementary Figure S2. Analyses of surface topography of GDH crystal. (**a**) The subunit images (cyan-coloured boxes) used for defining the *a*-axis (left panel) and *b*-axis (right panel) of the unit cell. (**b**) The positions of the selected subunit images (cyan-coloured boxes) used for analysing the surface height of N-domains.

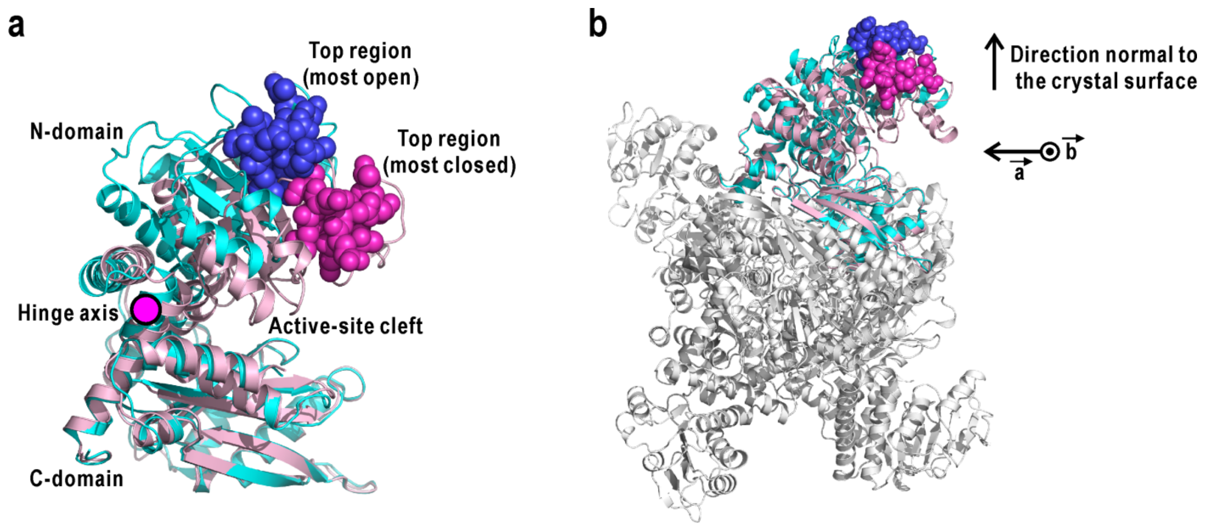

Supplementary Figure S3. The definitions of the tip of the N-domain (residues 277-287) of subunit D on the crystal surface. The locations of these illustrated by the CPK models in a subunit (**a**) and in subunit D of hexamer (**b**). In both panels, the locations are shown for the most open (cyan) and most closed (magenta) conformations.

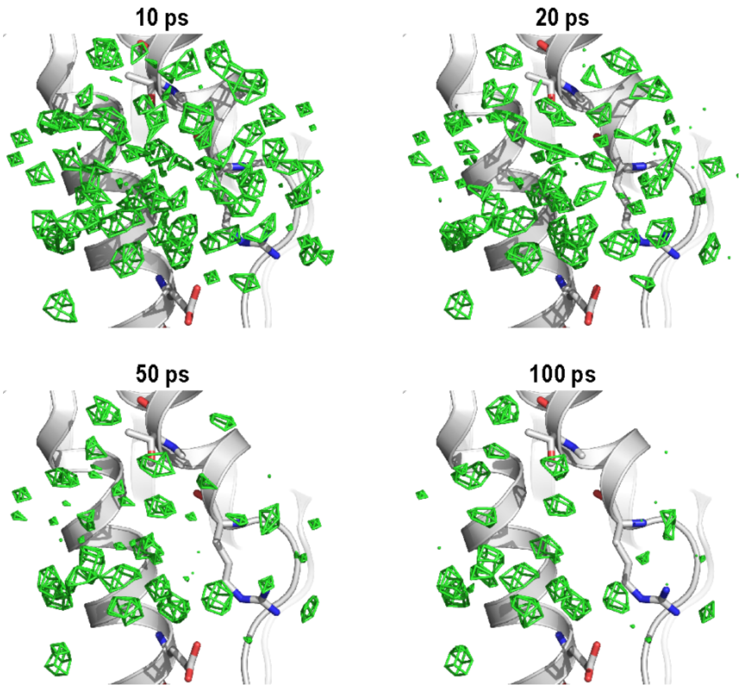

Supplementary Figure S4. Solvent density maps calculated using time windows of 10, 20, 50, and 100 ps (green fishnets). Each calculation was conducted on the snapshots starting from 140 ns in the trajectory of subunit A. The solvent density maps are 1.5 times of the average bulk solvent density.

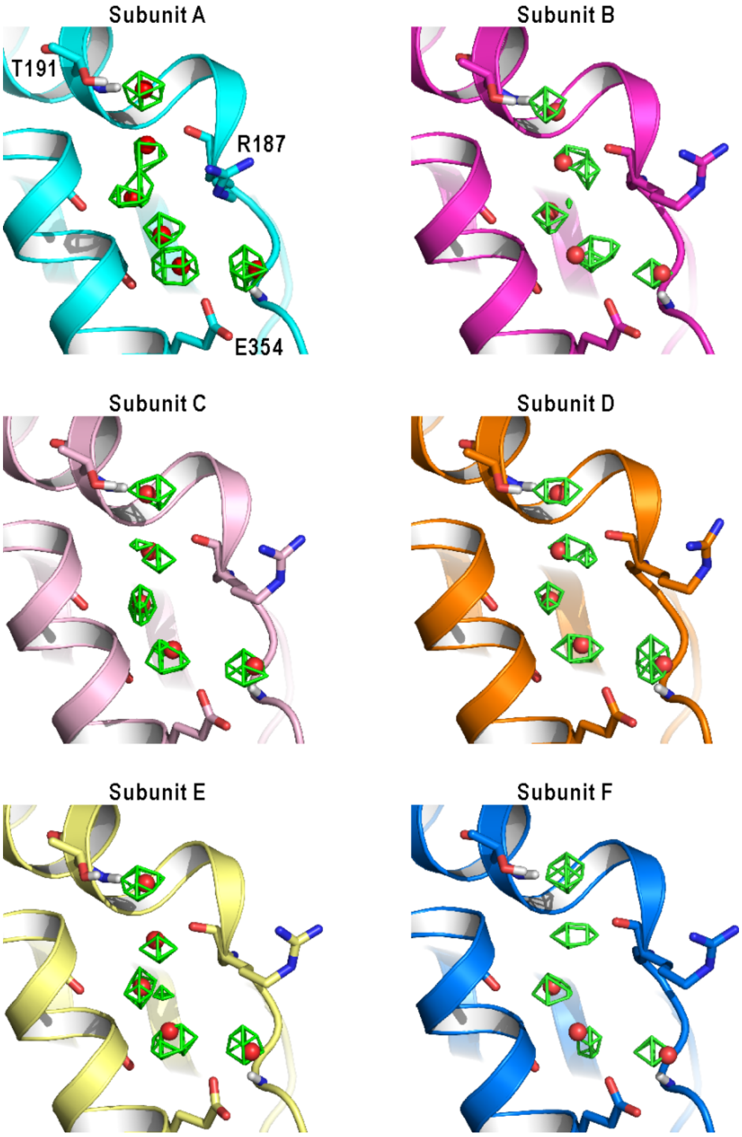

Supplementary Figure S5. Comparison of the water position from the crystal structure[22,23] (red sphere) and the peak positions in the solvent density maps (green fishnets) at the HS2 of subunits A-F. The solvent density maps were calculated from the last 50 ps of the solvent equilibration run. The solvent density maps are contoured at 1.5 times of the average bulk solvent density. In subunit F of the crystal structure, hydration water molecules are missed in the upper two hydration sites (indicated by arrows) of the crevice, because of the fluctuation of the N-domain.

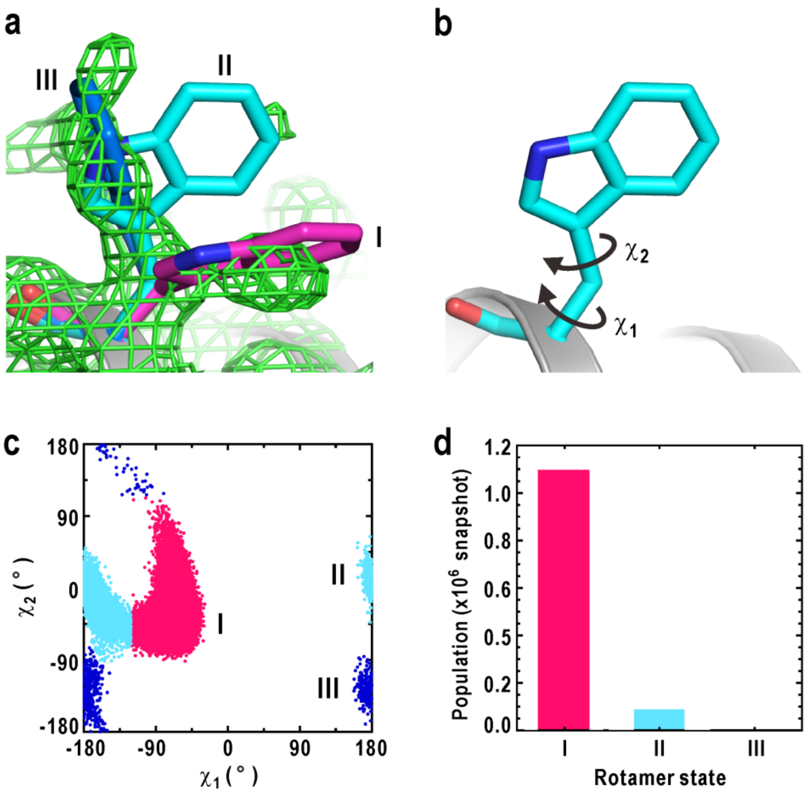

Supplementary Figure S6. Conformational variety of the sidechain of W89 at HS1. (**a**) Comparison of the electron density map (green fishnets) in the crystal structure[22,23] with the representative structures of rotamers I (pink sticks), II (cyan), and III (blue) of W89 in the MD simulation. The electron density map is the composite omit $2F_o - F_c$ difference Fourier electron density maps contoured at 1.0 standard deviation level from the average. The rotamers are defined by the χ_1 and χ_2 angles (**b**). (**c**) The distribution of rotamers in the $\chi_1 - \chi_2$ plane for snapshots sampled at every 50 ps in the trajectory. (**d**) The number of snapshots of six subunits belonging to each rotamer state during the 200-ns production run.

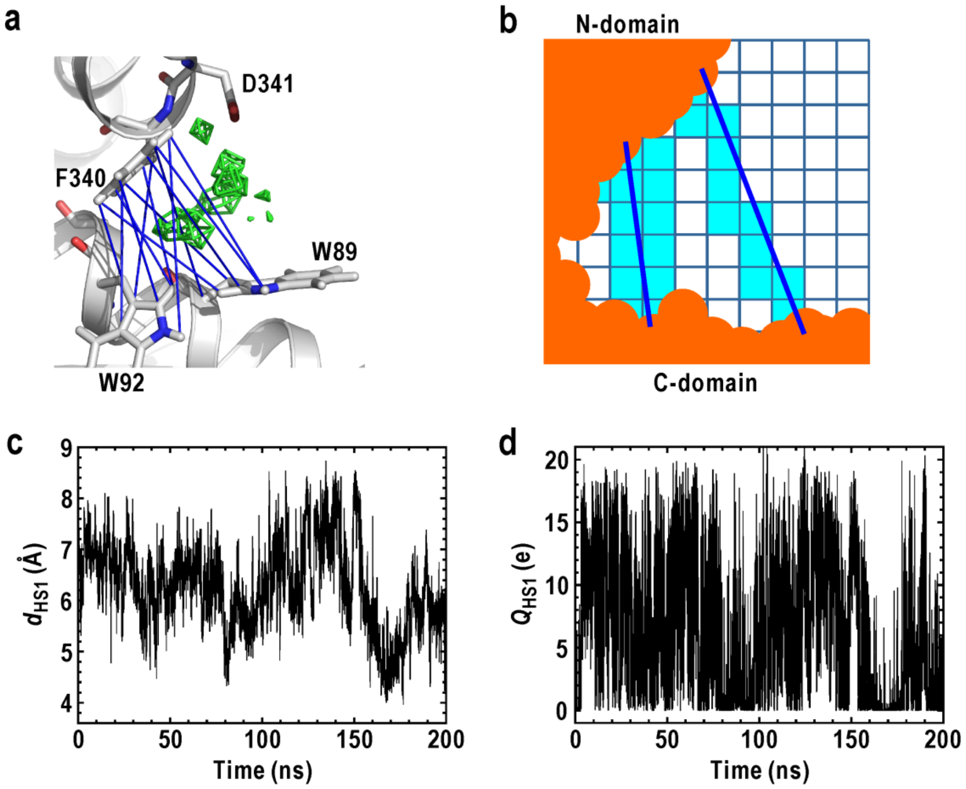

Supplementary Figure S7. Definitions of d_{HS1} and Q_{HS1}. (**a**) An examples of a set of line segments (blue lines) for the definition of the inside of the hydrophobic pocket at HS1. (**b**) A schematic illustration of 1-Å cube voxels judged to be located inside the hydrophobic pocket of HS1. The time courses of d_{HS1} (**c**) and Q_{HS1} (**d**) in the 200-ns MD trajectory of subunit A.

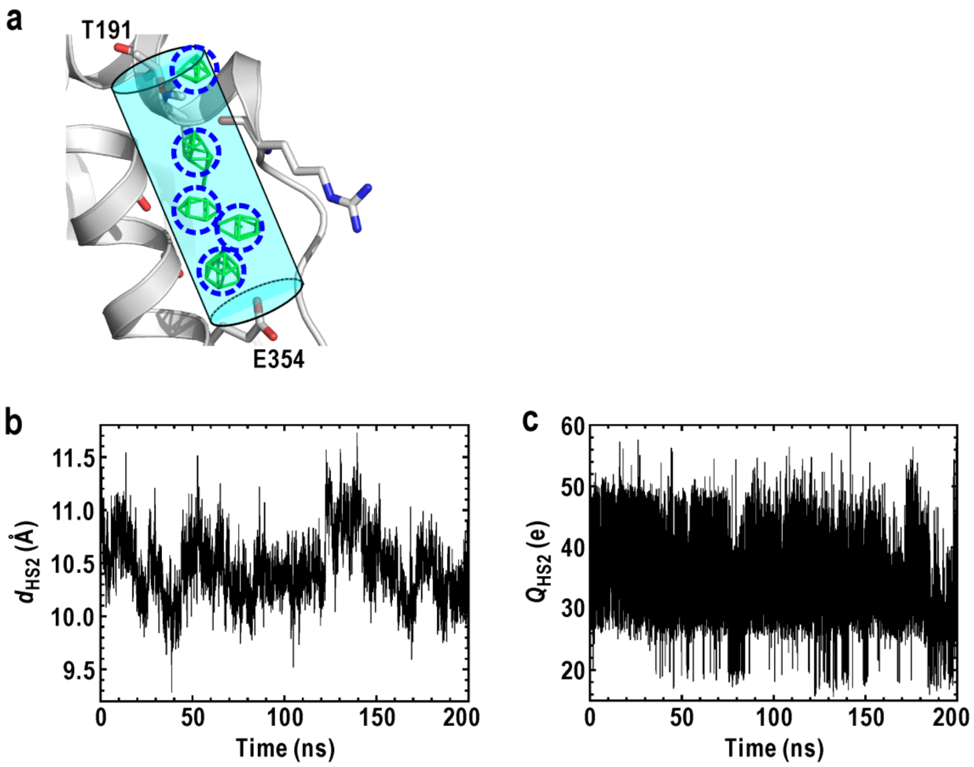

Supplementary Figure S8. Definitions of d_{HS2} and Q_{HS2}. (**a**) A cylinder approximating the shape of the hydrophilic crevice at HS2. The time courses of d_{HS2} (**b**) and Q_{HS2} (**c**) in the 200-ns MD trajectory of subunit A.

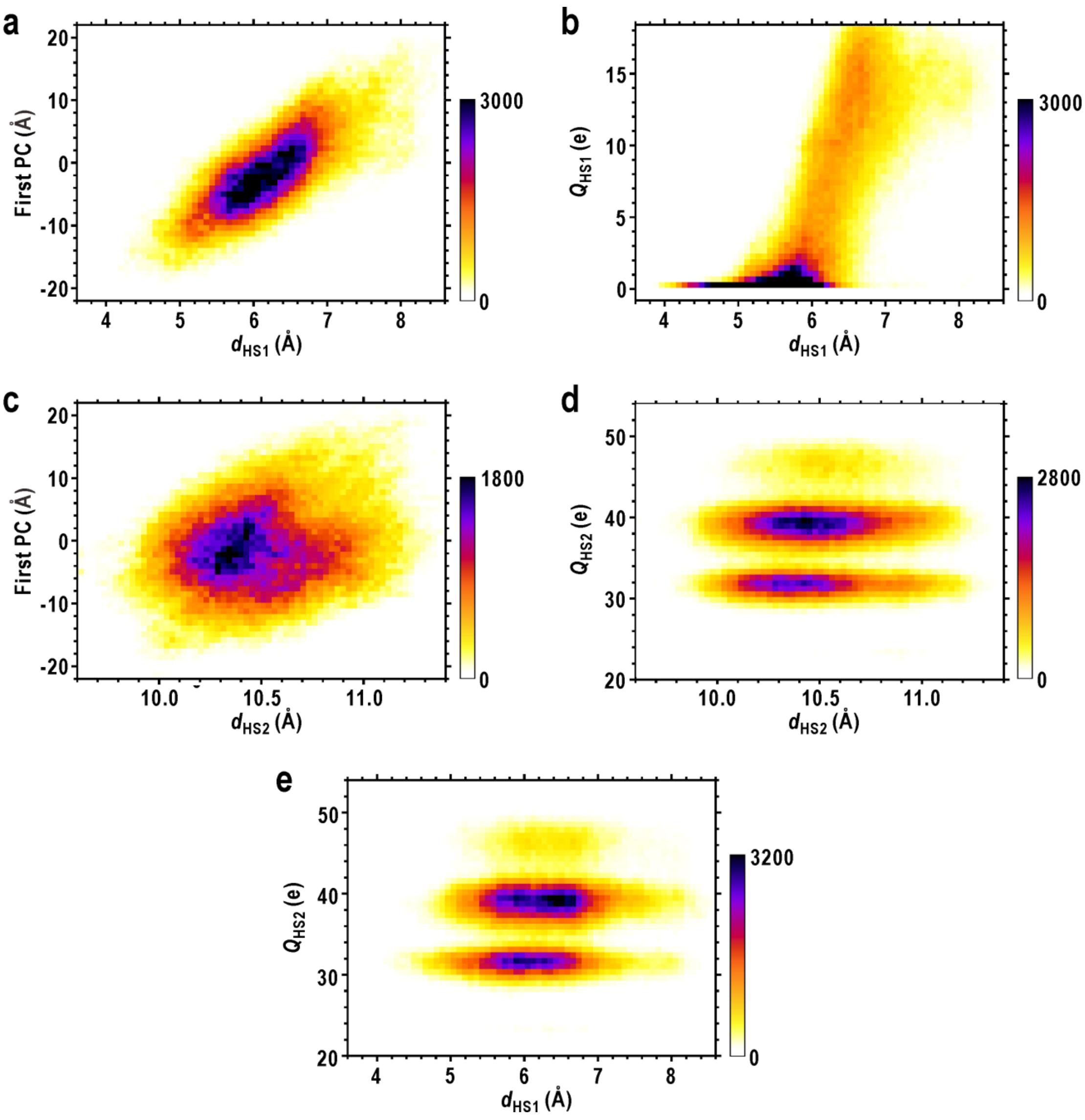

Supplementary Figure S9. The correlation plots among the structural and hydration parameters, d_{HS1}, Q_{HS1}, d_{HS2} and Q_{HS2}, calculated from the MD trajectories of all subunits. The heat maps show the relations between d_{HS1} and the N-domain motion represented by the first PC (**a**), between d_{HS1} and Q_{HS1} (**b**), between d_{HS2} and the first PC (**c**), between d_{HS2} and Q_{HS2} (**d**), and between d_{HS1} and Q_{HS2} (**e**).

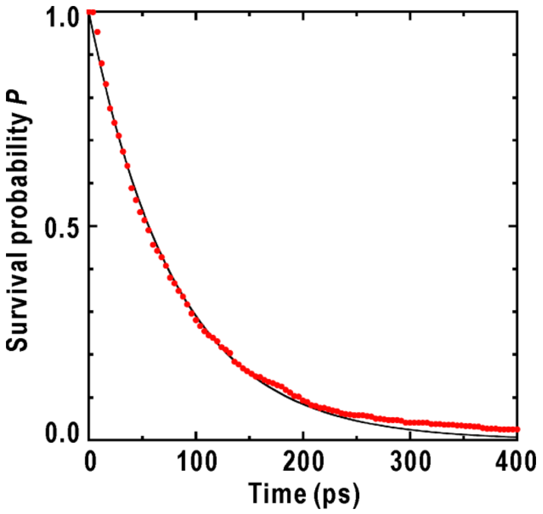

Supplementary Figure S10. The survival probability for the change from dry half-open to wet half-open states in HS1 (red dots) fitted by a model function (black line).

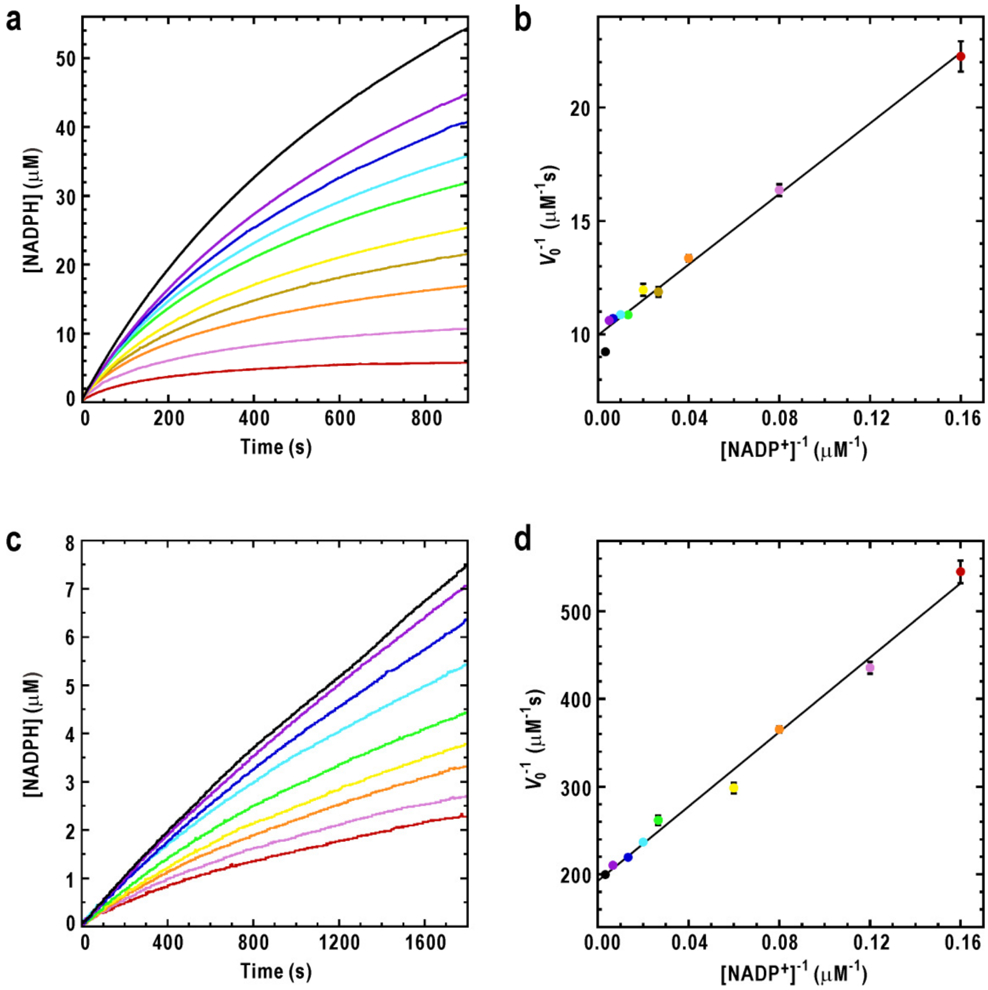

Supplementary Figure S11. The enzymatic activities of the wild-type and W89F-mutatant at 293 K. (**a**) The reaction curve for wild-type. The reaction curves are measured for the solutions containing 6.3 (red), 12.5 (pink), 25.0 (orange), 37.5 (dark-yellow), 50.0 (yellow), 75.0 (green), 100.0 (cyan), 150.0 (blue), 200.0 (purple), and 300.0 (black) μM $NADP^+$. (**b**) The dependence of V_0 on the concentration of $NADP^+$ for wild-type. (**c**) The reaction curves of W89F mutant in the reaction solutions containing 6.3 (red), 8.3 (pink), 12.5 (orange), 16.7 (yellow), 25.0 (green), 37.5 (cyan), 75.0 (blue), 150.0 (purple), and 300.0 (black) μM $NADP^+$. (**d**) The dependence V_0 on $NADP^+$ concentration for W89F mutant.

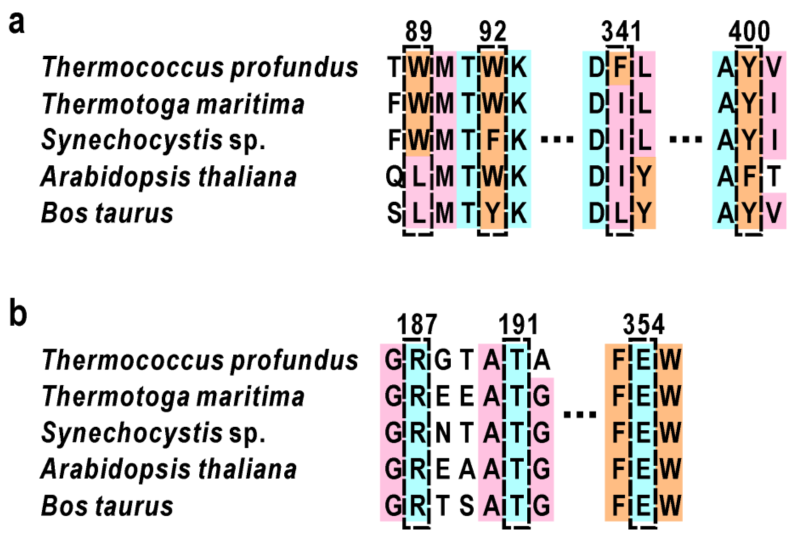

Supplementary Figure S12. A multiple alignment of members of the GDH family are shown. The residues belonging to HS1 (**a**) and HS2 (**b**) are enclosed in a frame. The orange, pink and cyan backgrounds indicate the aromatic, hydrophobic and hydrophilic residues, respectively, which are highly conserved among the family. The members of the family were identified by using PSI-BLAST[51]. The alignment was conducted by using CLUSTAL-W[52].

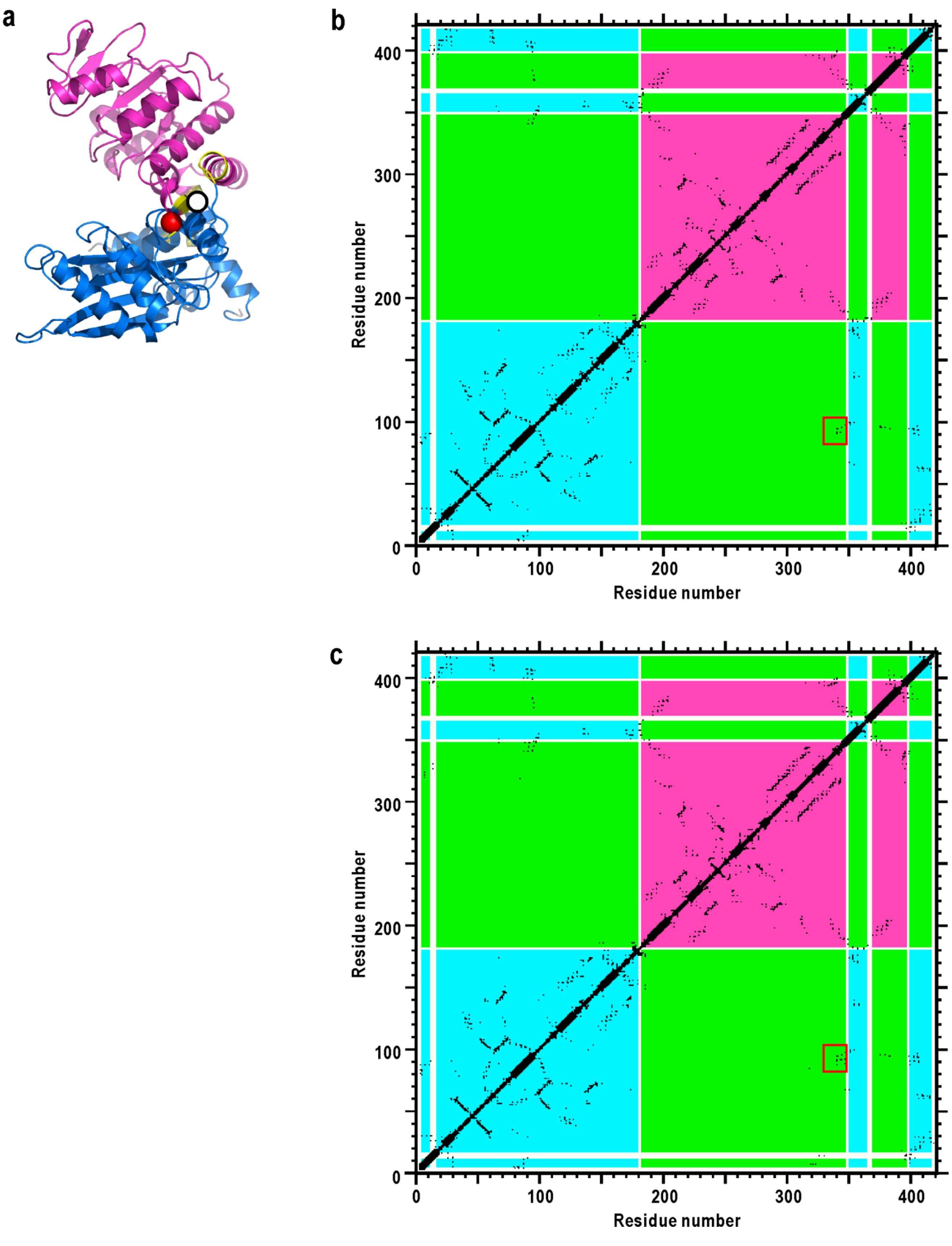
a
b
c
Residue number
Residue number
Residue number
Residue number
0
100
200
300
400

Supplementary Figure S13. Analyses on the changes in inter-residue interactions along with the domain motion from the most open to the most closed conformations. (**a**) C-domain (cyan-coloured region), N-domain (magenta) and hinge-bending regions (yellow) defined by the DynDom analysis[46,47]. The hinge axis and a hinge residue G183 are indicated by the white circle and the red sphere, respectively. (**b**) A contact map displaying the inter-residue interactions in the most open conformation. The intra-domain contacts within C- and N-domains are in the cyan- and magenta-coloured areas, respectively. The inter-domain contacts between the domains are in the green area. The contacts with the hinge-bending regions are in the narrow and white areas. The red box indicates the inter-domain contacts at HS1 (the contacts between residue groups 89-100 and 341-348). (c) A contact map displaying inter-residue interactions in the most closed conformation illustrated as panel (b).

Supplementary Table S1. K_m and k_{cat} values of Wild type and W89F mutant of GDH

Protein	K_m /μM	k_{cat} (s^{-1})
Wild type	7.8 ± 0.4	1.2 ± 0.0
W89F	11.0 ± 0.6	(6.1 ± 0.2) × 10^{-2}

Supplementary Table S2. Dihedral angles of the hinge residue, G183, in the most open and closed conformation

Conformations	φ (°)	ψ (°)
The most open	-110.0 [a] ± 26.8 [b]	171.4 ± 18.4
The most closed	-167.9 ± 12.4	-140.4 ± 14.5

[a] The shown dihedral angles were the averages calculated from a 50-ps time window around each conformation.

[b] The fluctuations of these dihedral angles were also calculated from the same time window.

Supplementary note 1: Analyses of surface topography of GDH crystal

Here we describe the procedures for analysing the measured surface topography in the following four subsections. We selected images composed of the unit cell arrays displaying almost identical structural features with a resolution to resolve individual subunits. Images were first corrected regarding the tilt of crystal surface against the plane movement of the piezo-scanner of the AFM equipment. Then, we carried out the assignment of unit cells followed by the calculation regarding the height distribution of N-domains in specified subunits. In addition, we describe the procedure to calculate the simulated height distribution of N-domain from the MD trajectory.

Supplementary note 1.1: Assignment of unit cells and N-domains

Because the N-domains of subunits D were found to be free from crystal contacts and protruded from the surface with the largest heights, these domains were used as an experimental reference to examine the magnitude of domain motions in the MD simulation. Several pairs of images of subunit D were selected (Supplementary Fig. S2a) to calculate the positions and dimensions of unit cells from topography images. In each selected subunit image, we detected peak region of 29×29 $Å^2$ (a box of 6×3 pixels) and calculated the centroids of this region. Then, the vectors connecting the centroids were defined for each pair of images (Supplementary Fig. S2a). From the lengths of the vectors, the unit cell dimensions of the surface lattice were calculated to be a = 129 Å, b = 170 Å, and γ = 90°. They were slightly larger than those determined by X-ray diffraction experiment for GDH crystal at ambient

temperature (a = 112.48 Å and b=163.12 Å) prior to the AFM measurement.

Supplementary note 1.2: Measured height distribution of N-domain motion

We used the height distribution of N-domain in subunit D to describe the conformational variety of GDH on crystal surface. An image of subunit D in each unit cell was masked by using a rectangular box of 15×5 pixels corresponding to 73×49 $Å^2$ (Supplementary Fig. S2b). We scanned each masked region using a box of 5×1 pixels (24×10 $Å^2$) to find which area had the largest height. Finally, we obtained the height distribution for the selected areas (Fig. 3e), the total number of which was 133 from three topographies.

To estimate the influences of the Brownian motion of cantilever probe at the tip[53,54] on the surface profile, we also measured surface topography of haemoglobin crystal[30]. We obtained a height distribution of haemoglobin subunits exposed to solvent from 99 images of the unit cells with the dimensions of 98×82 $Å^2$ (Fig. 2f) The obtained standard deviation of the distribution was used as a reference of the Brownian motion of a cantilever probe for analysing the height distribution of N-domains in D subunits of GDH. It should be noted that the viscosity of the stabilization buffer for haemoglobin crystal (1.5 M ammonium sulphate, 0.35 M ammonium phosphate, and 50 mM sodium cacodylate (pH 6.4)) was similar to that of the stabilization buffer for GDH crystal.

Supplementary note 1.3: Simulated height distribution of N-domain on crystal surface from MD trajectory

To compare the domain motion in the MD trajectory with the AFM results, we superimposed the C-domains of subunits in all trajectories onto the C-domain of the most exposed subunit in the unit cell of crystal surface (Supplementary Fig. S3). We first superimposed the C-domain of each subunit in 1.2×10^6 snapshots (the 200-ns trajectory × 6 subunits) of the MD trajectory onto the C-domain of subunit D in the unit cell of the crystal (Supplementary Fig. S3b). In each snapshot, we calculated the height h_{T} of the centroid of residues 277-287 at the tip of the N-domain of subunit D along the direction normal to the crystal surface. The height distribution (Fig. 3g) was presented as the deviation from the average $\langle h_{\mathrm{T}} \rangle_{\mathrm{MD}}$ for all snapshots as $h_{\mathrm{T}} - \langle h_{\mathrm{T}} \rangle_{\mathrm{MD}}$.

Supplementary note 2: Time window to calculate the solvent density map

Here we describe why we used a time window of 50 ps to calculate the solvent density map from the MD trajectory. Supplementary Fig. S4 illustrates the variety of solvent density maps calculated by using the different sizes of time windows. Peaks in the maps calculated using time windows of 50 and 100 ps are consistent with the crystal water sites[22,23]. In contrast, due to the noise in maps of 10 and 20 ps, it was difficult to identify the hydration sites in which water molecules resided stably. Thus, we used a time-window of 50 ps as the minimum period to reduce the noisy densities in solvent density maps.

Supplementary note 3: Comparison of the hydration structures between the solvent equilibration

run and the crystal structure

To examine whether the solvent density maps calculated from the MD trajectory reproduce hydration structures found in crystal structure[22,23], we compared the solvent density maps calculated from the 1.2-ns solvent equilibration run with the crystal water sites. Because we could not identify water molecules from the disordered electron density maps at HS1 in the crystal structure, the comparison was conducted only for HS2. The calculated solvent density maps at HS2 for the six subunits were consistent with the water positions in the crystal structure (Supplementary Fig. S5). The consistency strongly suggests that the current MD simulation, using the MARBLE software with the CHARMM27 force-field[42] and the TIP3P water model[40], is good for the investigation of the hydration structure in the cleft region.

Supplementary note 4: Conformational variety of the sidechain of W89 at HS1

In the crystal structure[22,23], subunit A had the most open conformation of the active-site cleft among the six subunits. The W89 sidechain of HS1 in subunit A displayed a disordered electron density map suggesting the conformational variety of more than two (Supplementary Fig. S6a). One of the conformations occupies the hydrophobic pocket and prevents both the closing movement of the N-domain and the penetration of water molecules into the hydrophobic pocket of HS1. Because this conformation is out of interest in this study, we surveyed the population of possible rotamers in the MD

trajectory, prior to the analysis for the hydration state at HS1.

In the trajectory of 200-ns run, we identified three rotamers designated as I, II and III with respect to χ_1 and χ_2 angles of W89 (Supplementary Fig. S6b,c). In rotamer I, hydration water molecules freely penetrate into HS1, while rotamers II and III prevent the penetration. The populations of rotamers I, II, and III were approximately 92, 7 and 0.3 %, respectively (Supplementary Fig. S6d). Therefore, we could analyse a huge number of snapshots of rotamer I in the analysis of hydration state of HS1 after excluding the snapshots of minor and unnecessary rotamers II and III. The exclusion of these rotamer states had little influences on our present results because of their small populations.

Supplementary note 5: Four parameters, d_{HS1}, Q_{HS1}, d_{HS2} and Q_{HS2}, to monitor hydration and structure changes at HS1 and HS2

To monitor the hydration structure changes along with the conformational changes at HS1 and HS2, we calculated the time courses of four parameters, d_{HS1}, Q_{HS1}, d_{HS2} and Q_{HS2}. Here we describe the details of the calculations.

The size of the hydrophobic pocket of HS1 along the direction of the N-domain motion was represented by parameter d_{HS1}, the distance between the $H_{\delta1}$ or $H_{\varepsilon1}$ atom of F340 in the upper jaw of the active-site cleft and the midpoint of the $C_{\delta1}$ atom of W89 and the C_γ atom of W92 in the lower jaw. Parameter Q_{HS1} monitored the amount of hydration water molecules residing in the hydrophobic pocket

as the sum of the solvent densities of only 1-Å cube voxels inside the hydrophobic pocket of HS1. To judge whether a voxel is inside the pocket, we used line segments drawn between atoms of the upper jaw (all atoms of F340) and of the lower jaw (C_α, C_β, C_γ, $C_{\delta1}$ atoms of W89 and all side-chain atoms of W92) (Supplementary Fig. S7a). When a voxel was located within 1.4 Å from any line segment, that voxel was judged to be inside of the pocket (Supplementary Fig. S7b).

The size of the crevice in HS2 was measured by parameter d_{HS2}, the distance between the A190 C_α atom and the E354 $O_{\varepsilon1}$ atom. Parameter Q_{HS2} reported the amount of hydration water molecules inside the crevice of HS2 as the sum of the solvent densities calculated through the following procedure. We first approximated the shape of the crevice by a cylinder with the radius of 2.5 Å (Supplementary Fig. S8a). The length of the cylinder is variable depending on the N-domain motion. The cylinder was set to the crevice so that its principal axis coincided with the line segment connecting the C_β atom of T191 and the O_ε atom of E354. We detected high solvent densities within the cylinder. Then, Q_{HS2} was calculated as the sum of the solvent densities in 1-Å cube voxels located within 1.7 Å from any centre positions of high solvent densities.

The time-courses of the four parameters were calculated for the six subunits throughout the 200-ns MD trajectory. Supplementary Fig. S9 compiles mutual dependences among the parameters. The results for the six subunits are almost consistent with those for subunit A shown in Figs. 6-8 in the main text.

Supplementary note 6: Enzymatic activities of wild type and W89F mutant of GDH

To investigate the effects of the hydrophobicity of HS1 on the function of GDH, the enzymatic activities of the wild-type and W89F-mutated GDH at 293 K were determined by measuring the reaction to reduce $NADP^+$ to NADPH[55] (Supplementary Fig. S11). The reaction mixture contained 100 mM Na phosphate buffer (pH 7.0), 10 mM sodium L-glutamate, 6.25-300 μM $NADP^+$, and 4 μg/ml GDH (49). The amount of the product, NADPH, was measured by monitoring the absorption at 340 nm with a spectrophotometer U-2900 (Hitachi High Technologies, Japan). The initial velocity of catalysis, V_0, was calculated from linear least-square fitting of the progress curves at the initial stages within 50 and 300 s for wild type and W89F, respectively. To estimate a catalytic rate, k_{cat}, and dissociation constant, K_m, the dependence of V_0 on $NADP^+$ concentration was analysed by the Michaelis-Menten equation. The resultant values of these parameters are listed in Table S1.

Supplementary note 7: Analyses on changes in inter-domain interactions along with the domain motion

To investigate the changes in inter-domain interactions along with the domain motion, we analysed the inter-residue interactions in the most open and closed conformations by using contact maps. The patterns of the inter-residue interactions within each domain and between the two domains can be easily observed by the contact maps (Supplementary Fig. S13). The most open and closed conformations were

identified according to the projection values of the snapshots on the first PC. Prior to the contact map analyses, dynamic domains and hinge-bending regions were defined by the DynDom analysis[46,47] for the two conformations (Supplementary Fig. S13a). Two dynamic domains were identified by the analysis as follows; dynamic domain 1 composed of residues 7-13, 19-181, 351-365 and 400-417, and dynamic domain 2 of residues 184-348 and 370-397. They are almost the same with C-domain and N-domain defined in the crystal structure[22]. The defined hinge-bending region is composed of residues 14-18, 182-183, 349-350, 366-369 and 398-399, which are located between the two domains.

In calculating the contact maps, we searched pairs of residues in contact. When the distance between any two non-hydrogen atoms belonging to different residues was less than 4.5 Å, the residues were defined to be in contact. There was little significant inter-domain interactions except those at HS1 in both the most open and closed conformations (Supplementary Fig S13b,c). At HS1, the increase in the number of inter-domain interactions was observed upon the domain closure, reflecting the structural packing of the hydrophobic pocket described in the main text. These results indicate that the domain motions of subunits are free from reorganization of inter-domain interactions, which causes an energy barrier for the motion.

We also analysed the dihedral angles of the residues belonging to the hinge-bending regions in the most open and closed conformations. Significant changes in dihedral angles between the two conformations were found only in G183. G183 is in the loop connecting C- and N-domains and near

HS2 (Table S2). Therefore, G183 is identified as the hinge residue for the domain motion according to the protocol of DynDom[47]. This result implies that the domain motion from the most open to the closed conformations require little changes in the secondary structures as well as those from RMSD analyses of the MD trajectories (Supplementary Fig. S1b).

Through these analyses, the conformational energy barrier for the structural changes from the open to closed conformations is probably small. This would be one of the reasons why relatively small changes in hydration at HS1 and HS2 have a profound impact on the large domain motion.

Supplementary Reference

51. Altschul, S. F. *et al.* Gapped BLAST and PSI-BLAST: a new generation of protein database search programs. *Nucleic. Acids. Res.* **25**, 3389–3402 (1997).

52. Thompson, J. D., Higgins, D. G. & Gibson T. J. CLUSTAL W: improving the sensitivity of progressive multiple sequence alignment through sequence weighting, position-specific gap penalties and weight matrix choice. *Nucleic. Acids. Res.* **22**, 4673–4680 (1994).

53. Engel, A., Schoenenberger, C. A. & Müller, D. J. High resolution imaging of native biological sample surfaces using scanning probe microscopy. *Curr. Opin. Struct. Biol.* **7**, 279-284 (1997).

54. Yokokawa, M., *et al.* Fast-scanning atomic force microscopy reveals the ATP/ADP-dependent conformational changes of GroEL. *EMBO. J.* **25**, 4567-4576 (2006).

55. Kobayashi, T., Higuchi, S., Kimura, K., Kudo, T. & Horikoshi, K. Properties of glutamate dehydrogenase and its involvement in alanine production in a hyperthermophilic archaeon, *Thermococcus profundus*. *J Biochem* **118**, 587-592 (1995).
